# Salp Navigation and Competitive based Parrot Optimizer (SNCPO) for efficient extreme learning machine training and global numerical optimization

**DOI:** 10.1038/s41598-025-97661-5

**Published:** 2025-04-21

**Authors:** Oluwatayomi Rereloluwa Adegboye, Afi Kekeli Feda, Ghanshyam G. Tejani, Aseel Smerat, Pankaj Kumar, Ephraim Bonah Agyekum

**Affiliations:** 1 University of Mediterranean Karpasia, Mersin-10, TR-10 Mersin, Mersin, Northern Cyprus Turkey; 2 Advanced Research Centre, European University of Lefke, TR-10 Mersin, Lefke, Northern Cyprus Turkey; 3 Department of Industrial Engineering and Management, Yuan Ze University, Taoyuan, 320315 Taiwan; 4 Applied Science Research Center, Applied Science Private University, Amman, 11937 Jordan; 5 Faculty of Educational Sciences, Al-Ahliyya Amman University, Amman, 19328 Jordan; 6 Centre for Research Impact & Outcome, Chitkara University Institute of Engineering and Technology, Chitkara University, Rajpura, Punjab 140401 India; 7 Department of Electrical and Electronics Engineering, Manipal Institute of Technology, Manipal Academy of Higher Education, Manipal, Karnataka 576104 India; 8 Department of Nuclear and Renewable Energy, Ural Federal University Named After the First President of Russia Boris Yeltsin, 19 Mira Street, Ekaterinburg, 620002 Russia; 9 Tashkent State University of Economics, Islam Karimov Street 49, 100066 Tashkent City, Uzbekistan; 10 Western Caspian University, Istiglaliyyat Street, Baku, Azerbaijan

**Keywords:** Parrot optimizer (PO), Competitive swarm optimization (CSO), Salp swarm algorithm (SSA), Extreme learning machine (ELM), Engineering, Electrical and electronic engineering

## Abstract

Metaheuristic optimization algorithms play a crucial role in solving complex real-world problems, including machine learning parameter tuning, yet many existing approaches struggle with maintaining an effective balance between exploration and exploitation, leading to premature convergence and suboptimal solutions. The traditional Parrot Optimizer (PO) is an efficient swarm-based technique; however, it suffers from inadequate adaptability in transitioning between exploration and exploitation, limiting its ability to escape local optima. To address these challenges, this paper introduces the Salp Navigation and Competitive based Parrot Optimizer (SNCPO), a novel hybrid algorithm that integrates Competitive Swarm Optimization (CSO) and the Salp Swarm Algorithm (SSA) into the PO framework. Specifically, SNCPO employs a pairwise competitive learning strategy from CSO, which divides the population into winners and losers. Winners are refined using SSA-inspired salp navigation, enabling enhanced global search in the early stages and a dynamic transition to exploitation. Meanwhile, losers are updated using PO’s communication strategy, reinforcing solution diversity and exploration. To validate the efficacy of SNCPO, rigorous experimental evaluations were conducted on CEC2015 and CEC2020 benchmark functions, four engineering design optimization problems, and Extreme Learning Machine (ELM) training tasks across 14 datasets. The results demonstrate that SNCPO consistently outperforms existing state-of-the-art algorithms, achieving superior convergence speed, solution quality, and robustness while effectively avoiding local optima. Notably, SNCPO exhibits strong adaptability to diverse optimization landscapes, reinforcing its potential for real-world engineering and machine learning applications.

## Introduction

ELM has emerged as a transformative paradigm in machine learning, especially for training single-hidden-layer feedforward neural networks (SLFNs). The ELM is an exceptional artificial neural network characterized by its swift training duration and outstanding generalization capabilities. In contrast to traditional gradient-based methods that require iterative weight adjustments and parameter tuning^1,2^, ELM employs a non-iterative learning process where input weights and biases are assigned randomly, and output weights are analytically computed using techniques such as the Moore–Penrose inverse^3^. This approach drastically reduces training time while maintaining competitive performance, making ELM particularly attractive. The advantages and functionalities of ELM have been demonstrated in several applications, including image processing^4^, pattern recognition^5^, cancer detection^6^, classification^7^, and fault diagnosis^8^. However, despite its computational efficiency, ELM faces hyperparameter tuning, accuracy, and generalization challenges, limiting its performance in complex and high-dimensional tasks^9^. To tackle these limitations, researchers have increasingly turned to Metaheuristic Algorithms (MAs), which offer flexible and adaptive solutions for enhancing ELM performance^10^. Among the various MAs, swarm intelligence (SI) algorithms have gained significant attention. SI is a sophisticated artificial intelligence discipline that employs various metaheuristic strategies inspired by the self-organized, intelligent, and collaborative behaviors of diverse insects and animals to solve real-world problems^11^. SI approaches consist of several homogeneous individuals engaging with one another and their surroundings through basic protocols to achieve an optimal solution. The agents are not exceptionally proficient individually. Nonetheless, their collaboration enables them to address intricate challenges^12^. Common SI algorithms include Particle Swarm Optimization (PSO)^13^, Ant Colony Optimization (ACO)^14^, Whale Optimization Algorithm (WOA)^15^, Salp Swarm Algorithm (SSA)^16^, Jellyfish Search Optimizer (JSO)^17^, Manta Ray Foraging Optimization (MRFO)^18^, Artificial Bee Colony (ABC)^19^, Dragonfly Algorithm (DA)^20^, Grasshopper Optimization Algorithm (GOA)^21^, Harris Hawks Optimization (HHO)^22^, Lion Optimization Algorithm (LOA)^23^, Bat Algorithm (BA)^24^, Cuckoo Search Algorithm (CSA)^25^, Firefly Algorithm (FA)^26^, Artificial Gorilla Troops Optimizer (AGT)^27^, and the Parrot Optimizer (PO)^28^. While these algorithms have been applied in various domains and achieved competitive results, MAs are not without limitations. One major challenge is their susceptibility to premature convergence, wherein the algorithm becomes stuck in suboptimal solutions, particularly in highly multimodal or noisy search spaces^29,30^. Many MAs struggle to maintain a balance between exploration (searching for new solutions) and exploitation (refining existing solutions), often leading to either insufficient exploration or inefficient exploitation^31^. Another limitation is their sensitivity to parameter settings; poorly chosen parameters can drastically reduce the algorithm’s effectiveness, requiring extensive tuning and increasing computational costs^32^. These limitations are particularly pronounced in high-dimensional and complex problems^33,34^. In response to these limitations, researchers have introduced various enhancements to traditional MAs^35–38^.

The Parrot Optimizer (PO) is a recently proposed swarm intelligence-based optimization algorithm inspired by the communication and learning behaviors of parrots. Four main behaviors: foraging, staying, talking, and fear of strangers, seen in domesticated Pyrrhura Molinae parrots, are codified into four unique formulas to aid in the search for optimal solutions. Unlike typical metaheuristic algorithms, which have discrete exploration and exploitation phases, each individual in the PO population randomly chooses one of these four behaviors during each iteration. This strategy more accurately represents the behavioral unpredictability observed in domesticated Pyrrhura Molinae parrots while also dramatically increasing population diversity. Due to this non-adaptive optimization approach, PO exhibits certain weaknesses, including premature convergence and an imbalance between exploration and exploitation, particularly in complex, high-dimensional search spaces. This study addresses the limitations of PO by introducing the Salp Navigation and Competitive-based Parrot Optimizer (SNCPO) for ELM training and global numerical optimization. The SNCPO addresses these limitations by combining the exploratory strengths and dynamic transition to the exploitation phase of the SSA with the competitive learning solution refinement capabilities of the CSO. SNCPO achieves a synergistic effect that balances exploration and exploitation while maintaining scalability and adaptability by integrating these two strategies. The key contributions of this research are as follows:

1. Development of the SNCPO Algorithm: This study introduces the SNCPO algorithm by integrating the competitive learning strategy and the salp navigation mechanism. This integration of the salp navigation mechanism enhances the balance and adaptive transition between exploration and exploitation and facilitates local optima escape, as solution refinement through competitive learning effectively searches for better solutions, effectively addressing the limitations of the original PO.
2. Comprehensive Benchmark Evaluation: Rigorous empirical validation is conducted using standard benchmark functions, including the CEC2015 and CEC2020 test suites, to assess the optimization performance of SNCPO.
3. Application to Real-World Engineering Optimization Problems: The effectiveness of SNCPO is further validated through its application to four complex engineering design problems, showcasing its ability to produce high-quality solutions and demonstrating its potential for practical engineering applications.
4. Extensive Experimental Evaluation on ELM Training: A rigorous experimental study is performed to assess the performance of SNCPO in optimizing the parameters of the ELM. The algorithm is tested on 14 datasets.

The manuscript is structured as follows: Sect “Literature review” provides a literature review of the current state of research, and Sect “Materials and methods” covers materials and methods. Sect “Experiment and discussion” discusses the experiment and findings, followed by the conclusion and future work in Sect “Conclusion and future work”.

## Literature review

MAs, inspired by natural phenomena and biological processes, have demonstrated remarkable potential in optimizing various aspects of ELMs. Boriratrit et al. demonstrated the effectiveness of integrating algorithms with ELM to enhance forecasting precision. The study introduces a novel framework incorporating several algorithms. Using Thailand’s electric energy demand data, the proposed models were rigorously evaluated against state-of-the-art algorithms. The results show that Jellyfish Search Optimizer (JS) based ELM achieves superior accuracy, as demonstrated by the lowest root mean square error^39^. Wu et al.'s study investigates the performance of hybrid models combining the ELM with MAs, namely Whale Optimization Algorithm (WOA) and Flower Pollination Algorithm (FPA) for Pan Evaporation (Ep) prediction. The proposed WOA-ELM and FP-ELM models were compared with other ML models. Results demonstrated that FP-ELM achieved the highest accuracy^40^. Furthermore, Yang et al. proposed a novel method based on the ELM to extract unknown parameters of both electrochemical and simplified electrochemical solid oxide fuel cell models. Eight MAs were utilized for efficient parameter extraction. The performance of optimizer-based ELM was rigorously evaluated, and the results demonstrate the method’s effectiveness in achieving high accuracy, stability, speed, and robustness^41^. Also, Thieu et al. 's study addresses the challenges traditional models often encounter with nonlinearity, stochastic behavior, and convergence, specifically in accurate river streamflow prediction. The study proposes novel hybrid models combining ELM with advanced MAs, comprising the Runge–Kutta Optimizer (RUN), Weighted Mean of Vectors (INFO), and Pareto-like Sequential Sampling (PSS). Utilizing streamflow data, 20 hybrid models were evaluated. Results exhibited the superiority of mathematically inspired models in terms of accuracy, convergence, and stability. Notably, the PSS-ELM model attained the best performance^42^. Abba et al., optimized the ELM using four MAs: Genetic Algorithm (GA), PSO, Biogeography-Based Optimization (BBO), and a hybrid BBO-PSO to predict treated water quality parameters. Models performance was evaluated using several metrics. The BBO-PSO-ELM model demonstrated greater predictive performance compared to others^43^. Bacanin et al.’s. study addresses ELM’s limited performance due to reliance on the optimal tuning of weights and biases in the hidden layer, posing a continuous optimization challenge. To address this limitation, this study proposes a multi-swarm hybrid optimization approach that integrates three swarm intelligence MAs: the Firefly Algorithm (FA), Artificial Bee Colony (ABC), and Sine Cosine Algorithm (SCA). The novel method was rigorously assessed on seven benchmark classification datasets and compared against cutting-edge approaches. Results demonstrate superior generalization performance in terms of accuracy, precision, recall, and F1-score^44^. Dogan and Ozkan optimized the ELM model with the HHO and the PSO. The results demonstrated that the enhanced ELM models achieved superior stability and accuracy^45^.

More advanced research has applied hybrid or enhanced MAs, which integrate multiple algorithms or incorporate adaptive strategies to optimize ELM, thus reducing the susceptibility to premature convergence and improving the balance between exploration and exploitation. Zhong et al. introduced a hierarchical RIME algorithm featuring multiple search preferences aimed at addressing complex optimization problems to overcome the drawbacks of the original RIME algorithm, such as preserving population diversity and preventing premature convergence. The proposed algorithm divides the population into inferior, borderline, and superior layers according to their fitness. Each layer employs distinct search operators. The authors conducted extensive experiments on engineering problems, benchmark functions, and ELM optimization. Statistical analyses confirmed the superiority of the novel RIME optimizer, demonstrating its scalability and applicability^46^. Heidari et al. proposed an improved Grey Wolf Optimizer (GWO), termed OBLGWO, to overcome the shortcomings of the conventional GWO in solving complex optimization problems. The authors addressed its susceptibility to local optima and suboptimal convergence by incorporating opposition-based learning, random spiral-form motions, levy flight patterns, and greedy selection to enhance exploration, exploitation, and search efficiency. The novel OBLGWO was rigorously assessed on benchmark functions and Kernel Extreme Learning Machine (KELM) tuning. The findings underscore the efficacy of OBLGWO as a robust optimization tool for complex, real-world problems^47^. Cao et al. addressed the limitations of the ELM, particularly the random generation of connection weights and bias during training, which can lead to complexity and reduced generalization ability. To overcome these challenges, the authors introduced an enhanced Crow Search Algorithm (CSA) for optimizing ELM parameters. They incorporated PSO to improve global search effectiveness. In the later stages of iteration, a Gaussian function with a penalty coefficient was introduced to induce local perturbations, adaptively adjust parameters, and avoid entrapment in local optima. The improved CSA was then applied to optimize the ELM weights, achieving accurate prediction results^48^. Cai et al. proposed a novel parameter tuning strategy for the KELM based on an Improved Grey Wolf Optimization (IGWO) algorithm. Recognizing the critical impact of model parameters on KELM performance, the authors introduced a hierarchical mechanism to enhance the stochastic behavior and exploration capability of grey wolves in the optimization process. Specifically, the strategy incorporated a random local search around the optimal grey wolf for Beta wolves and a random global search for Omega wolves, thereby achieving an improved balance between exploration and exploitation. Experimental findings highlight the potential of IGWO as an effective tool for optimizing KELM parameters, enabling it to achieve superior performance in real-world scenarios^49^. Liu et al. proposed a modified Parrot Optimizer (PO) for ELM parameter tuning^50^. The authors proposed a Normal Cloud Parrot Optimization (NCPO) algorithm, which integrates the cloud model with the flocking behavior of Pyrrhura Molinae parrots to improve exploration and exploitation. NCPO-ELM achieved superior forecasting accuracy for photovoltaic energy prediction.

Despite the advancements in MAs for optimizing ELM, several critical gaps persist, limiting their effectiveness in addressing the unique challenges posed by ELM optimization. One of the primary concerns is the scalability of existing MAs when applied to large datasets and high-dimensional features. Many traditional and enhanced optimization techniques struggle to maintain computational efficiency and solution quality as the problem complexity increases. Furthermore, a fundamental limitation of ELM arises from its random initialization of input weights and biases, which directly impacts model generalizability. This randomness often results in suboptimal parameter settings, leading to reduced predictive accuracy and inconsistency across different datasets. Current MAs, while effective in ELM training tasks, lack the adaptive mechanisms required to mitigate the effects of ELM’s inherent limitations and enhance its generalization capability.Additionally, most existing optimization approaches fail to achieve a dynamic balance between exploration and exploitation. While some algorithms prioritize extensive search (exploration) at the cost of slower convergence, others focus on rapid convergence (exploitation), increasing the risk of premature stagnation in local optima. This trade-off remains a persistent challenge in ELM optimization, necessitating the development of algorithms that can dynamically transition between global search and local refinement without compromising convergence speed or solution quality. A critical review of the literature reveals that while traditional and improved MAs have made significant strides in refining ELM optimization, existing approaches still suffer from limited generalizability, suboptimal prediction accuracy, and inefficient adaptation to high-dimensional tasks. These shortcomings underscore the need for advanced hybrid optimization strategies capable of adaptively balancing exploration and exploitation while enhancing ELM’s robustness across diverse problem domains.

Therefore, this research proposes SNCPO to address these gaps and the limitations of the traditional PO. The traditional PO suffers from several critical limitations that hinder its effectiveness in solving complex optimization problems. One of the primary challenges associated with conventional PO is the imbalance between exploration and exploitation, which often results in premature convergence and suboptimal solutions. The lack of an adaptive transition mechanism between these two phases significantly impairs the algorithm’s ability to escape local optima and conduct an efficient global search. Additionally, PO’s static search strategy fails to dynamically adjust to the evolving nature of the optimization landscape, thereby limiting its capacity to refine solutions effectively. To address these shortcomings, the SNCPO algorithm integrates the exploratory strengths and adaptive transition mechanisms of the SSA with the competitive learning-driven refinement capabilities of the CSO approach. SSA’s systematic, chain-based movement strategy enhances global exploration by ensuring a thorough search across the solution space, thereby mitigating the risks of premature convergence and increasing the likelihood of identifying globally optimal solutions. Furthermore, SSA’s adaptive transition mechanism enables a seamless shift from exploration to exploitation, enhancing the overall search efficiency. Simultaneously, CSO introduces a competitive learning paradigm that refines solutions through a population-based selection mechanism. By categorizing candidate solutions into winners and losers, CSO promotes selective updating strategies, where winners are refined using the salp navigation approach derived from SSA, while losers undergo enhancement via PO’s communication-based updating mechanism. This dual refinement strategy fosters diversity in the search process and prevents stagnation. With these complementary strategies, SNCPO achieves a synergistic balance between exploration and exploitation, effectively overcoming the limitations of traditional PO. The algorithm does not only enhance solution accuracy but also ensures adaptability and scalability across diverse and complex optimization problems, such as ELM parameter tuning. Consequently, SNCPO emerges as a robust and efficient optimization framework capable of addressing the complex challenges associated with modern optimization tasks. Although the study by Liu et al.^50^, has enhanced ELM using an improved PO, our research distinguishes itself by introducing a hybrid and adaptive approach. Specifically, our proposed SNCPO integrates a competitive learning strategy with an adaptive salp navigation mechanism, ensuring a more adaptive balance between exploration and exploitation.

## Materials and methods

### Parrot optimizer (PO)

The PO draws inspiration from the behavioral traits of Pyrrhura Molinae, a popular parrot species known for its sociability, trainability, and close bonding with owners. This parrot portrays four distinct behaviors namely: foraging, staying, communicating, and fear of strangers. These behaviors form the foundation of the PO algorithm. Foraging involves locating food in groups using visual and olfactory cues. Staying refers to random perching on an owner’s body. Communicating encompasses social interactions within the flock, while fear of strangers drives the birds to avoid unfamiliar individuals and seek safety with their owners. The stochastic nature of these behaviors motivates their integration into the PO framework^28^. The initial population of candidate solutions is determined randomly within predefined search space bounds. For a swarm size $N$, maximum iterations ${Max}_{\text{iter}}$, and search space limits $lb$ (lower bound) and $ub$ (upper bound), the position of the ${i}^{\text{th}}$ individual is initialized according to Eq. (1).

1 $${X}_{i}^{0}=lb+\text{rand}(\text{0,1})\cdot (ub-lb)$$

$rand(\text{0,1})$ denotes a uniformly distributed random number in the interval $[\text{0,1}]$, and ${X}_{i}^{0}$ represents the initial position of the ${i}^{\text{th}}$ individual. During foraging, individuals estimate food locations based on the host’s position. The positional update is given in Eq. (2).

2 $${X}_{i}^{t+1}=\left({X}_{i}^{t}-{X}_{\text{best }}\right)\cdot \text{Levy}(\text{dim})+\text{rand}(\text{0,1})\cdot {\left(1-\frac{t}{{\text{Max}}_{\text{iter }}}\right)}^{\frac{2t}{\text{ Maxiter }}}\cdot {X}_{\text{mean }}^{t}$$

where ${X}_{i}^{t}$ and ${X}_{i}^{t+1}$ denote the current and updated positions, respectively. ${X}_{\text{best}}$ is the best solution identified so far, ${X}_{\text{mean }}^{t}$ is the average position of the population, and $Levy(dim)$ models the flight pattern using the Levy distribution. The term $\left({X}_{i}^{t}-{X}_{\text{best }}\right)\cdot Levy(dim)$ simulates movement toward the host, while the second term incorporates group-level observations. The Levy distribution is computed as expressed in Eq. (3), (4), (5).

3 $$\text{Levy}(\text{dim})=\frac{\mu \cdot \sigma }{|v{|}^{\frac{1}{\gamma }}}$$

4 $$\mu ,v\sim N(0,\text{dim})$$

5 $$\sigma ={\left(\frac{\Gamma (1+\gamma )\cdot \text{sin}\left(\frac{\pi \gamma }{2}\right)}{\Gamma \left(\frac{1+\gamma }{2}\right)\cdot \gamma \cdot {2}^{\frac{1+\gamma }{2}}}\right)}^{\frac{1}{\gamma +1}}$$

Here, $\gamma =1.5$, and $\Gamma$ denotes the gamma function. The staying behavior models random perching on the owner’s body. The position update is given in Eq. (6).

6 $${X}_{i}^{t+1}={X}_{i}^{t}+{X}_{{\text{best}} \, }\cdot \text{Levy}(\text{dim})+\text{rand}(\text{0,1})\cdot \text{ones}(1,\text{dim})$$

Here, ones $(1,dim)$ is a vector of ones representing random stopping points. This equation combines flight toward the host ( ${X}_{\text{best }}\cdot Levy(dim)$ ) and random perching. Communication involves interaction within the flock. Two scenarios are modeled with equal probability: joining the flock or communicating without joining the flock. The update rule is expressed in Eq. (7).

7 $${X}_{i}^{t+1}=\left\{\begin{array}{ll}0.2\cdot \text{rand}(\text{0,1})\cdot \left(1-\frac{t}{{\text{ Max }}_{\text{ter }}}\right)\cdot \left({X}_{i}^{t}-{X}_{\text{mean }}^{t}\right),& P\le 0.5\\ 0.2\cdot \text{rand}(\text{0,1})\cdot \text{exp}\left(-\frac{t}{\text{rand}(\text{0,1})\cdot {\text{ Max }}_{\text{ter }}}\right),& P>0.5\end{array}\right.$$

Here, $P$ is an arbitrary number in $[\text{0,1}]$, and ${X}_{\text{mean }}^{t}$ denotes the flock’s center. To model avoidance of strangers, the update rule incorporates reorientation toward the host and distancing from strangers, as expressed in Eq. (8).

8 $${X}_{i}^{t+1}={X}_{i}^{t}+\text{rand}(\text{0,1})\cdot \text{cos}\left(0.5\pi \cdot \frac{t}{{\text{Max}}_{\text{iter }}}\right)\cdot \left({X}_{\text{best }}-{X}_{i}^{t}\right)-\text{cos}(\text{rand}(\text{0,1})\cdot \pi )\cdot {\left(\frac{t}{{\text{Max}}_{\text{iter }}}\right)}^{\frac{2}{{\text{maxter}}^{2}}}\cdot \left({X}_{i}^{t}-{X}_{\text{best}}\right)$$

### Proposed SNCPO

#### Salp navigation strategy

The SSA, proposed by Mirjalili et al.^16^, is a nature-inspired optimization algorithm that emulates the predation behavior of salp swarms. Salps are marine organisms that propel themselves through the water by ingesting it and forming chain-like structures during hunting to facilitate rapid population movement. In SSA, the salp swarm is separated into two roles: leaders and followers. The leader represents the individual at the forefront of the swarm, directing the group toward the prey while the followers come behind, adjusting their positions based on the leader’s movements. This hierarchical structure enables efficient exploration and exploitation of the search area. Leaders, positioned at the front of the swarm, adjust their position according to the position of the target (the best solution identified so far). The position update rule for the leaders in the ${j}^{\text{th}}$ dimension is given in Eq. (9).

9 $${X}_{j}^{t+1}=\left\{\begin{array}{ll}{X}_{j}^{best}+{\alpha }_{1}\cdot \left(\left(u{b}_{j}-l{b}_{j}\right)\cdot {\alpha }_{2}+l{b}_{j}\right),& {\alpha }_{3}\ge 0\\ {X}_{j}^{best}-{\alpha }_{1}\cdot \left(\left(u{b}_{j}-l{b}_{j}\right)\cdot {\alpha }_{2}+l{b}_{j}\right),& {\alpha }_{3}<0\end{array}\right.$$

Here, ${X}_{j}^{t+1}$ symbolizes the updated location of the leader in the ${j}^{\text{th}}$ dimension. ${X}_{j}^{best}$ represents the location of the target (best solution) in the ${j}^{\text{th}}$ dimension. $l{b}_{j}$ and $u{b}_{j}$ are the lower and upper bounds of the ${j}^{\text{th}}$ dimension, respectively. ${\alpha }_{2}$ and ${\alpha }_{3}$ are arbitrary numbers in the interval [0, 1]. ${\alpha }_{1}$ regulates the trade-off between exploration and exploitation, as shown in Eq. (10).

10 $${\alpha }_{1}=2{e}^{-{\left(\frac{4t}{\text{ Maxiter }}\right)}^{2}}$$

Here, $t$ is the current iteration, and ${Max}_{\text{iter}}$ is the maximum number of iterations. The exponential decay of ${\alpha }_{1}$ ensures a gradual shift from exploration (early iterations) to exploitation (later iterations). The position update rule for the ${i}^{\text{th}}$ follower in the ${j}^{\text{th}}$ dimension is given in Eq. (11):

11 $${X}_{j}^{t+1}=\frac{1}{2}\left({X}_{j}^{t}+{X}_{j}^{t-1}\right), i\ge 2$$

where ${X}_{j}^{t+1}$ designates the updated location of the ${i}^{\text{th}}$ follower in the ${j}^{\text{th}}$ dimension. The random parameters ${\alpha }_{2}$ and ${\alpha }_{3}$ in the leader update equation introduce stochasticity into the search process of SNCPO. This randomization allows the algorithm to explore portions of the search region that may otherwise remain unexplored, thereby boosting the likelihood of discovering globally optimal solutions. In SNCPO, this stochastic movement complements the interactions among search agents, preventing an early convergence of the population to suboptimal solutions. The exponential decay of ${\alpha }_{1}$ ensures a seamless transition from exploration to exploitation in SNCPO. During early iterations, when ${\alpha }_{1}$ is large, the leader explores more regions of the search space, increasing diversity. As ${\alpha }_{1}$ decreases over time, the leader focuses on exploiting promising regions near the best-known solution.

#### Competitive learning strategy

Competitive Swarm Optimization (CSO) is a recently developed variation of the PSO algorithm^51^. Unlike traditional PSO, which relies on personal best and global best locations to guide particle updates, CSO operates by retaining only the position ${X}_{i}^{t}=\left[{x}_{i,1}^{t},{x}_{i,2}^{t},\dots ,{x}_{i,D}^{t}\right]$ and velocity ${V}_{i}^{t}=\left[{v}_{i,1}^{t},{v}_{i,2}^{t},\dots ,{v}_{i,D}^{t}\right]$, here $t$ denotes the generation index, and $D$ represents the dimensionality of the problem^52^. In each generation, the population of $N$ particles is arbitrarily split into $N/2$ groups, with each group consisting of two particles. Among these pairs, the particle with better fitness is designated as the winner, while the other is labeled as the loser. The winners are carried over to the next generation without modification, whereas the losers undergo an update process that incorporates learning from the winners. This mechanism ensures that the population evolves through competition, promoting diversity and effective exploration. The losers are updated as mathematically expressed in Eqs. (12), (13).

12 $$V_{l}^{t + 1} = R_{1} \cdot V_{l}^{t} + R_{2} \cdot \left( {X_{w}^{t} - X_{l}^{t} } \right) + R_{3} \cdot \phi \cdot \left( {\overline{X}^{t} - X_{l}^{t} } \right)$$

13 $$X_{l}^{t + 1} = X_{l}^{t} + V_{l}^{t + 1}$$

$l$ and $w$ designate the indices of the loser and winner particles, respectively. ${X}_{l}^{t}$ and ${V}_{l}^{t}$ denote the location and velocity of the loser at generation $t$. ${X}_{w}^{t}$ is the location of the corresponding winner particle. $\overline{X}^{t}$ is the average location of the whole population at generation $t$, $\phi$ is a parameter that regulates the impact of the population’s average position $\overline{X}^{t}$ on the loser’s movement. ${R}_{1},{R}_{2}$, and ${R}_{3}$ are uniform random numbers determined within the interval [0, 1]. The selection of winners and losers in the CSO algorithm is a pivotal step that governs the competitive interactions within the population. This process involves a pairing mechanism to ensure unbiased comparisons between individuals. The winner of the pair is determined based on the fitness values of the two individuals randomly chosen from the population. Mathematically, this is expressed as seen in Eq. (14).

14 $$\text{Winner }\left(m\right)=\left\{\begin{array}{ll}{p}_{1},& \text{ if fitness }\left({p}_{1}\right)<\text{ fitness }\left({p}_{2}\right)\\ {p}_{2},& \text{ otherwise}\end{array}\right.$$

fitness $\left({p}_{1}\right)$ and fitness $\left({p}_{2}\right)$ represent the objective function values of the respective particles randomly selected from the search population. The individual with the better (lower) fitness value is designated as the winner. The loser of the pair is determined as expressed in Eq. (15).

15 $$\text{Loser}(m)=\left\{\begin{array}{ll}{p}_{2},& \text{ if fitness }\left({p}_{1}\right)<\text{ fitness }\left({p}_{2}\right)\\ {p}_{1},& \text{ otherwise}\end{array}\right.$$

In this case, the individual with the inferior (higher) fitness value is labeled as the loser. The proposed SNCPO approach splits the individuals into two sets: winners and losers, based on fitness evaluations. This competitive learning mechanism ensures that only the fittest individuals (winners) directly influence the evolution of the population, while the less fit individuals (losers) undergo guided updates to improve their positions. By splitting the population, the algorithm introduces a dual-layered optimization process. Winners represent the elite solutions and are updated using the leader strategy from SSA, which promotes exploration and efficient transition to exploitation. Losers are updated using the communication strategy from PO to promote exploration and communication within the population. This division ensures that the model simultaneously focuses on refining promising solutions and exploring new portions of the search space.

**Algorithm 1:**
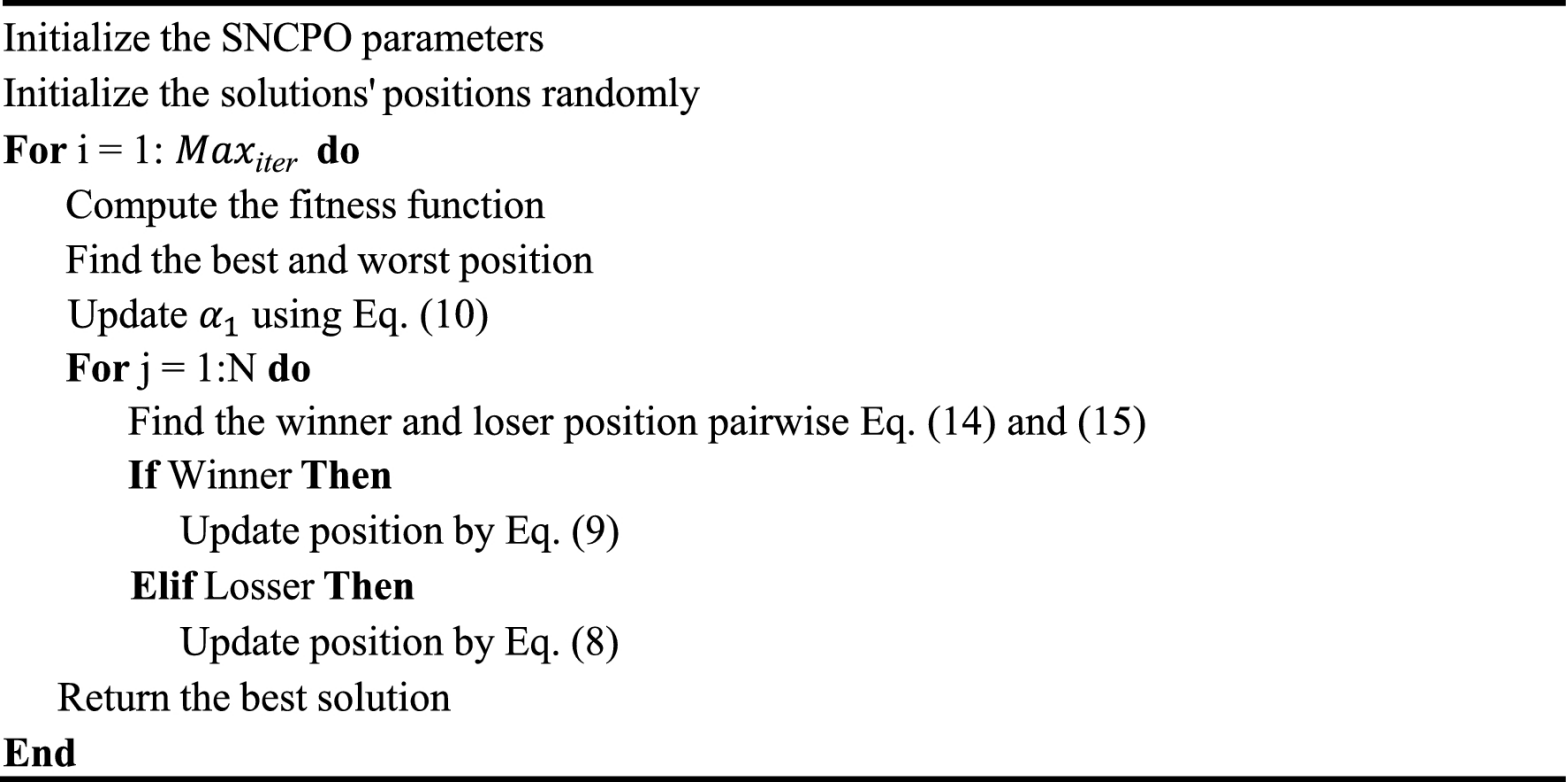
Pseudo-code of the SNCPO algorithm.

**Fig. 1:**
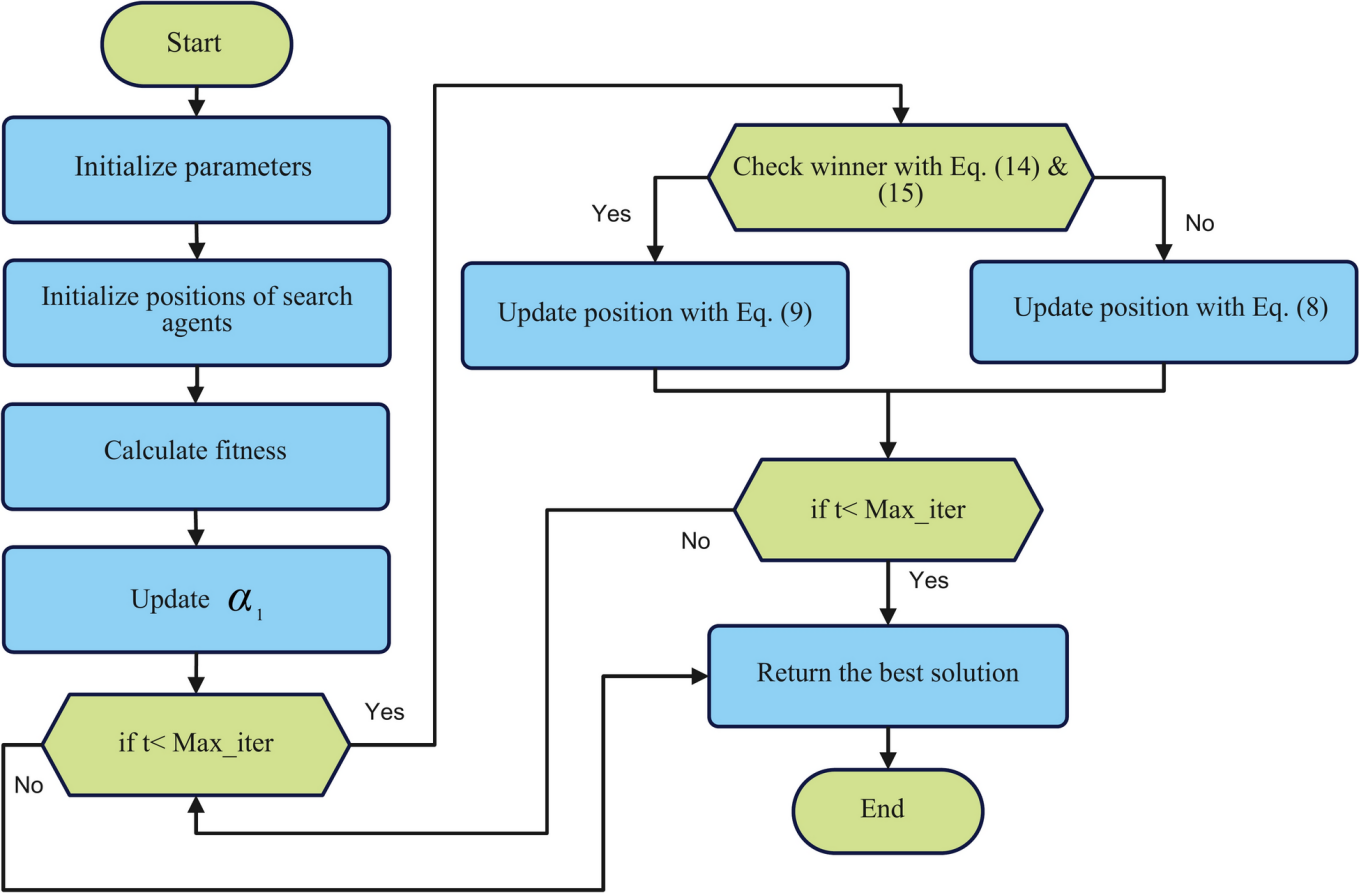
SNCPO flowchart.

As seen in Algorithm 1, SNCPO initializes its parameters at the initial stage, which include population size $N$, maximum iterations ${Max}_{\text{iter,}}$, and control parameters such as ${\alpha }_{1}$. The optimization process proceeds iteratively for ${Max}_{\text{iter}}$ iterations, during which the fitness function is computed for each solution to assess its quality, and the best and worst positions are identified to guide the search process. A key feature of SNCPO is its competitive learning mechanism, where the population is randomly paired, and individuals are classified as winners or losers based on their fitness values using pairwise comparison using a permutation list as expressed in Eq. (14) and (15). Winners representing elite solutions are updated using the leader update mechanism from the SSA, as seen in Eq. (9), which refines their positions near the best-known solution to enhance exploitation and exploration. The ${\alpha }_{1}$ parameter dynamically adjusts the transition from exploration as iteration increases. In the early stages, winners can efficiently search the problem space for near-global solution regions; as iteration reaches the maximum, winners can gradually refine their solutions. Conversely, losers, representing less fit solutions, are updated using the PO communication strategy, as seen in Eq. (8), which promotes exploration by encouraging diverse movements within the search space. Upon completing all iterations, the algorithm returns the best solution found, representing the optimal or near-optimal position in the search space. Combining competitive learning with SSA’s salp navigation and PO strategies, this hybrid approach enables SNCPO to successfully address complex optimization problems by leveraging the complementary strengths of exploration and exploitation, adaptively transitioning between exploration and exploitation, compared to the traditional PO, which randomly applies different strategies to the population. Thereby ensuring strong performance across various problem domains. The Flowchart of SNCPO is illustrated in Fig. 1.

#### Computation complexity of SNCPO

The computational complexity of the SNCPO algorithm can be analyzed by examining the key operations performed during its execution. Initially, the algorithm requires $O(N\cdot D)$ computations for the random initialization of $N$ solutions within a $D$-dimensional problem space. During each iteration, the fitness function is evaluated for all $N$ solutions, incurring a cost of $O(N\cdot F)$, where $F$ represents the computational cost of evaluating the fitness function for a single solution. Additionally, the pairwise competition process involves generating a random permutation of the population and performing fitness-based comparisons, which collectively require $O(N)$ operations. The position updates for winners and losers, guided by the salp navigation and PO strategies, respectively, involve arithmetic operations and random number generation, contributing $O(N\cdot D)$ computations per iteration. Since these steps are repeated for ${Max}_{\text{iter}}$ iterations, the total computational complexity of SNCPO is $O(\left.{\text{Max}}_{\text{iter }}\cdot N\cdot (F+D)\right)$.

### ELM training

#### Original ELM

**Fig. 2:**
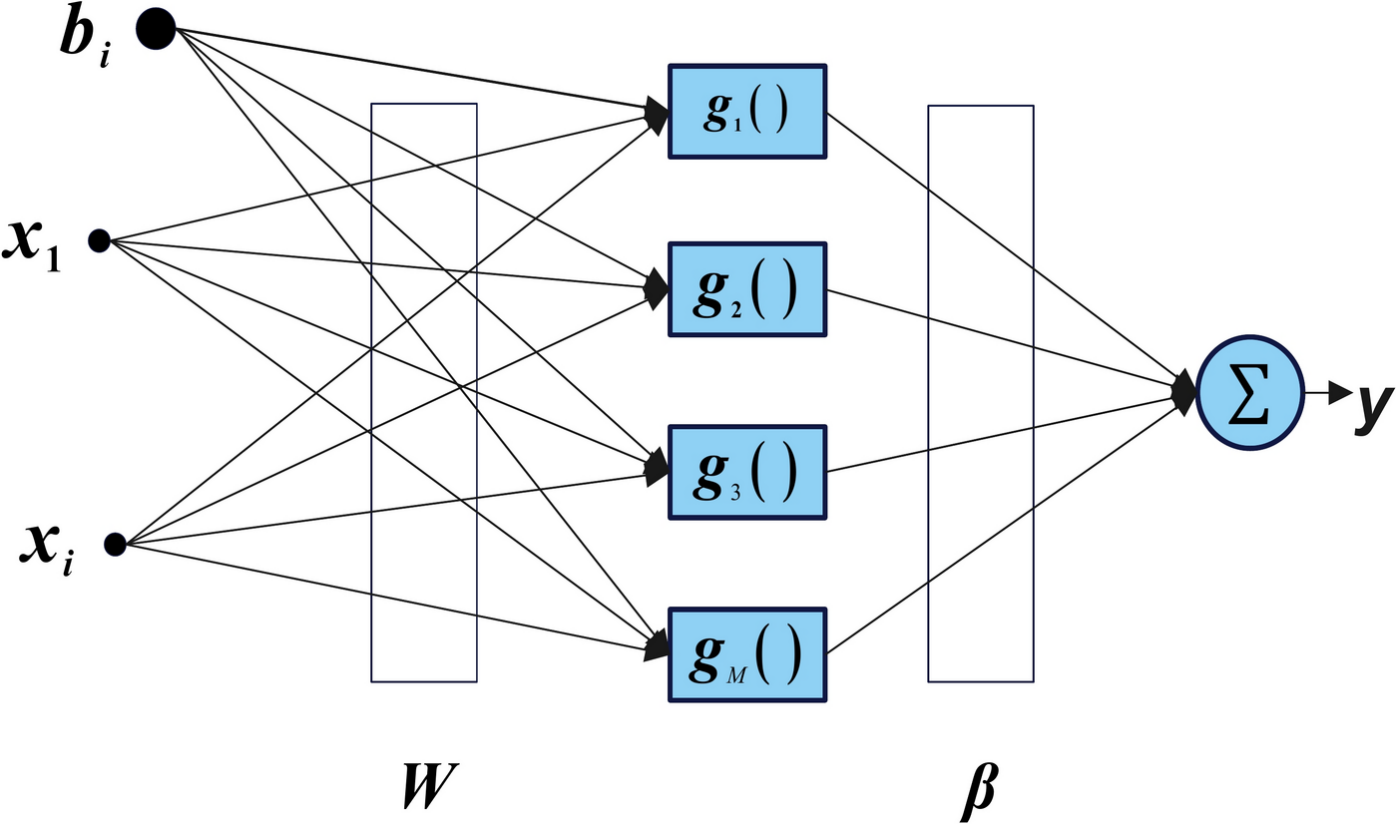
ELM model.

The ELM is a feed-forward neural network characterized by its single hidden layer, which has demonstrated exceptional performance in both regression and classification tasks^3^. Unlike traditional neural network-based methods, ELM eliminates the need for iterative tuning of hidden layer parameters. Instead, the weights and biases of the input layer neurons are arbitrarily assigned^45^. The activation functions for the hidden layer neurons can include Sigmoid, ReLU, or Tanh functions, while a linear activation function is typically employed for the output layer neurons. As illustrated in Fig. 2, ELM’s architecture comprises three layers: the input, hidden, and output. For an ELM network with $M$ neurons in the hidden layer and $N$ samples, the output of the model can be mathematically expressed as given in Eq. (16).

16 $${o}_{j}=\sum_{i=1}^{M} {\beta }_{i}g\left({w}_{i}\cdot {x}_{j}+{b}_{i}\right), j=\text{1,2},\dots ,N$$

where ${o}_{j}$ denotes the output vector of the ELM. ${\beta }_{i}$ denotes the output weights linking the hidden layer to the output layer. $g(\cdot )$ is the activation function applied to the hidden layer neurons. ${w}_{i}$ represents the input weights connecting the input layer to the hidden layer. ${b}_{i}$ is the bias value associated with the ${i}^{\text{th}}$ hidden neuron. ${x}_{j}$ is the ${j}^{\text{th}}$ input sample. The hidden layer output matrix $H$ can be calculated as defined in Eq. (17) from Eq. (16).

17 $$H=g\left({w}_{i}\cdot {x}_{j}+{b}_{i}\right)$$

Then, Eq. (17) can be converted into matrix form, as shown in Eq. (18).

18 $$H\beta =y$$

There $H$ is the hidden layer output matrix, explicitly defined in Eq. (19).

19 $$H={\left[\begin{array}{ccc}g\left({w}_{1}\cdot {x}_{1}+{b}_{1}\right)& \cdots & g\left({w}_{M}\cdot {x}_{1}+{b}_{M}\right)\\ \vdots & \ddots & \vdots \\ g\left({w}_{1}\cdot {x}_{N}+{b}_{1}\right)& \cdots & g\left({w}_{M}\cdot {x}_{N}+{b}_{M}\right)\end{array}\right]}_{N\times M}$$

$\beta$ which is the output weight vector is expressed in Eq. (20)

20 $$\beta ={\left[\begin{array}{c}{\beta }_{1}\\ \vdots \\ {\beta }_{M}\end{array}\right]}_{M\times 1}$$

$y$ which is the target output vector is defined in Eq. (21)

21 $$y={\left[\begin{array}{c}{y}_{1}\\ \vdots \\ {y}_{N}\end{array}\right]}_{N\times 1}$$

In the ELM approach, the input weights ${w}_{i}$ and biases ${b}_{i}$ are arbitrarily allocated, and the output weights $\beta$ are computed using the generalized inverse Moore–Penrose (MP) matrix of $H$. Specifically, the output weights $\hat{\beta }$ are calculated as given in Eq. (22)

22 $$\hat{\beta } = H^{\dag } y$$

where $\hat{\beta }$ is the approximate output weight vector. $H^{\dag }$ is the Moore–Penrose generalized inverse of the hidden layer output matrix $H$.

#### Proposed SNCPO-ELM model

The individual vectors within the population used for optimization in SNCPO-ELM model are constructed as a combination of input weights $(w)$ and hidden biases (b). The proposed SNCPO is employed to calculate the optimal input weights and biases, with the goal of enhancing the performance of the ELM classifier on several datasets. The search for the best solution occurs within a $(n\times H+H)$ dimensional search space, where $n$ denotes the number of input features, and $H$ represents the number of hidden neurons. Both ${w}_{i}$ (input weights) and ${b}_{i}$ (hidden biases) are randomly initialized within the range $[-\text{10,10}]$. For each individual in the SNCPO population, the corresponding output weights are calculated, and the fitness of each individual is subsequently assessed. The fitness of each individual is evaluated based on the classification accuracy achieved on the training set. Accuracy is a widely used metric in classification studies and measures the proportion of correct predictions relative to the overall number of samples. It is mathematically expressed Eq. (23)

23 $$\text{ACC}=\frac{{T}_{P}+{T}_{N}}{{T}_{P}+{T}_{N}+{F}_{P}+{F}_{N}}\times 100$$

**Fig. 3:**
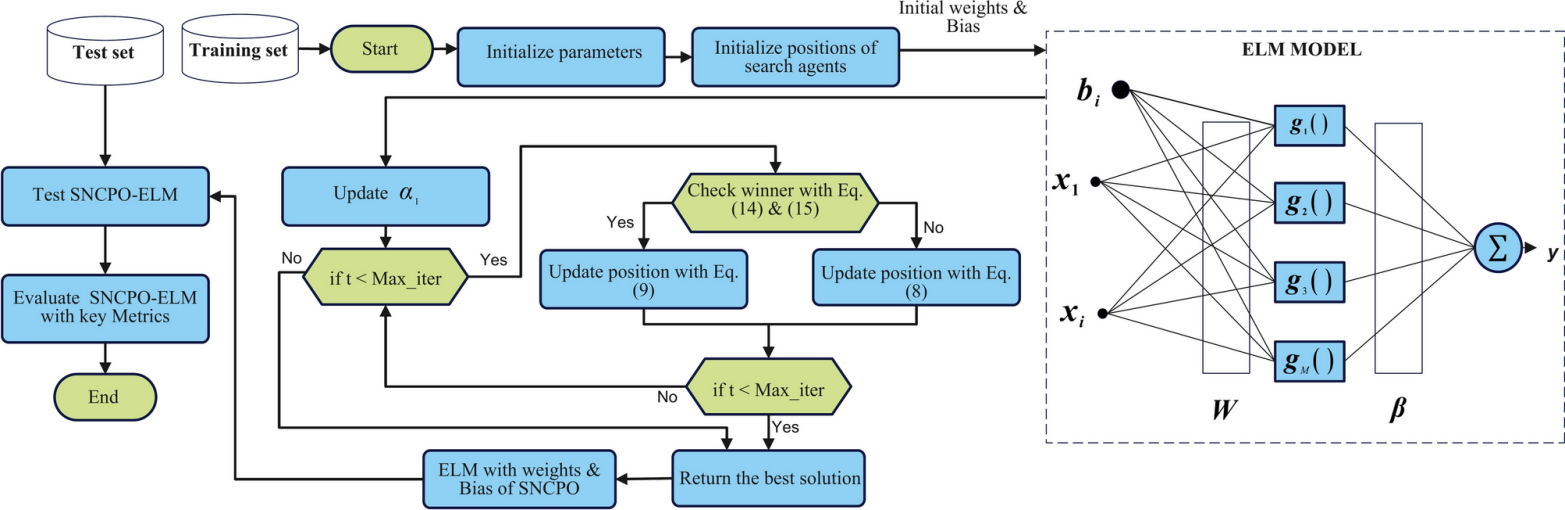
SNCPO-ELM model.

Here ${T}_{P}$ denotes the true positives and represents correctly predicted positive samples. ${T}_{N}$ represents true negatives, representing correctly predicted negative samples. ${F}_{P}$ is false positives, representing incorrectly predicted positive samples. While ${F}_{N}$ is false negatives, represents incorrectly predicted negative samples. The SNCPO-ELM model is shown in Fig. 3.

## Experiment and discussion

To validate the robustness and credibility of the experimental results, this study exclusively utilizes the CEC 2015^53^ and CEC 2020^54^ benchmark suites. These benchmark functions are systematically classified into distinct categories. For the CEC 2015 suite, the functions are grouped as follows: F1-F2 represent unimodal functions, F3-F5 denote simple multimodal functions, F6-F8 correspond to hybrid functions, and F9-F15 encompass composition functions. Similarly, the CEC 2020 test suite comprises F16, which includes single-modal functions; F17-F19, representing basic multimodal functions; F20-F22, grouped as hybrid functions; and F23-F25, which consists of composition functions. The parameter configurations for each algorithm used in the experiment for comparison are comprehensively outlined in Table 1. Key experimental parameters have been carefully defined to maintain consistency and fairness in the evaluation process. These include a maximum iteration limit of 5000, 30 independent runs for statistical reliability, a problem dimensionality of 30, and a population size of 30. The proposed SNCPO algorithm is benchmarked against a set of comparative algorithms, which are as follows: Parrot Optimizer (PO)^28^, Aquila Optimizer (AO)^55^, Exponential Distribution Optimizer (EDO)^56^, Grey Wolf Optimizer (GWO)^57^, Moth Flame Optimization (MFO)^58^, Opposition Based Learning Path Finder Algorithm (OBLPFA)^59^, Sine Cosine Algorithm (SCA)^60^. This rigorous setup ensures that the performance evaluations are both comprehensive and rigorous.

**Table 1:** Algorithm parameters.

Algorithm	Parameter
PO	$\alpha$= rand[0,1]/5, $\theta$ = rand[0,1] *$\pi$
AO	µ = 0.00565, $\omega =0.005,$ $\alpha \text{ = }\delta =0.1$
EDO	Switch parameter = 0.5
GWO	${a}_{0}=2$
MFO	b = 1, a = [-2, -1]
OBLPFA	$\alpha , \beta =[1, 2]$
SCA	$a=2$
SNCPO	${\alpha }_{1}=[2/e,2]$

### Statistical and convergence analysis of compared optimizers on the CEC2015

To comprehensively evaluate the efficacy of the SNCPO algorithm, an extensive experiment using the CEC2015 benchmark functions is conducted, comparing its performance against several state-of-the-art optimization algorithms. The experimental results, as summarized in Table 2, illustrate the superior performance of SNCPO across multiple optimization functions. In Table 2, the best results obtained for each function are highlighted in bold, underscoring the effectiveness of SNCPO in solving complex optimization tasks. A thorough analysis of Table 2 reveals that the SNCPO algorithm consistently achieves near-optimal solutions across a wide range of benchmark functions, significantly outperforming the original PO algorithm. This superior performance can be attributed to SNCPO’s ability to maintain a well-balanced interplay between global exploration and local exploitation, which is a critical factor in achieving optimal solutions. Additionally, Table 2 presents key performance evaluation metrics, including the average (AVG) and standard deviation (STD) values, providing valuable insights into the comparative performance of SNCPO relative to other optimization approaches.

**Table 2:** Experimental result of SNCPO and other optimizers on CEC2015.

		SNCPO	PO	AO	EDO	GWO	MFO	OBLPFA	SCA
F1	AVG	**1.74E + 4**	4.30E + 10	4.19E + 10	9.84E + 9	1.32E + 9	7.30E + 9	2.01E + 9	1.23E + 10
	STD	**5.52E + 4**	5.48E + 9	6.22E + 9	4.50E + 9	1.42E + 9	4.10E + 9	2.99E + 8	3.02E + 9
F2	AVG	**1.34E + 3**	6.04E + 4	5.78E + 4	3.35E + 4	2.48E + 4	8.93E + 4	6.48E + 4	3.47E + 4
	STD	**5.18E + 2**	5.13E + 3	3.59E + 3	7.43E + 3	6.89E + 3	3.54E + 4	2.58E + 4	5.49E + 3
F3	AVG	3.28E + 2	3.39E + 2	3.42E + 2	3.29E + 2	**3.16E + 2**	3.26E + 2	3.28E + 2	3.36E + 2
	STD	4.63	**1.43**	2.46	3.63	2.32	4.05	2.99	2.61
F4	AVG	**4.36E + 3**	7.64E + 3	6.84E + 3	6.89E + 3	4.71E + 3	5.45E + 3	7.92E + 3	7.67E + 3
	STD	5.86E + 2	3.42E + 2	8.32E + 2	5.88E + 2	2.04E + 3	7.24E + 2	3.71E + 2	**3.04E + 2**
F5	AVG	**5.01E + 2**	5.03E + 2	5.02E + 2	5.03E + 2	5.03E + 2	5.01E + 2	5.03E + 2	5.03E + 2
	STD	**2.48E−1**	2.81E−1	4.35E−1	3.94E−1	2.70E−1	6.85E−1	3.97E−1	2.71E−1
F6	AVG	6.01E + 2	6.05E + 2	6.04E + 2	6.03E + 2	**6.00E + 2**	6.01E + 2	6.01E + 2	6.02E + 2
	STD	1.60E−1	3.08E−1	2.58E−1	9.60E−1	**9.09E−2**	9.07E−1	1.82E−1	4.24E−1
F7	AVG	**7.01E + 2**	7.82E + 2	7.61E + 2	7.27E + 2	7.01E + 2	7.21E + 2	7.02E + 2	7.24E + 2
	STD	**3.23E−1**	1.36E + 1	5.73	9.43	3.21	1.89E + 1	9.60E−1	2.99
F8	AVG	**8.19E + 2**	3.77E + 6	1.86E + 6	5.85E + 4	1.28E + 3	7.74E + 4	1.44E + 3	3.62E + 4
	STD	**8.54**	1.77E + 6	1.01E + 6	1.64E + 5	1.43E + 3	1.35E + 5	7.20E + 2	2.21E + 4
F9	AVG	9.12E + 2	9.13E + 2	9.13E + 2	9.13E + 2	**9.12E + 2**	9.13E + 2	9.13E + 2	9.13E + 2
	STD	4.01E−1	**1.54E−1**	2.48E−1	2.23E−1	6.24E−1	3.65E−1	1.90E−1	2.29E−1
F10	AVG	**2.90E + 5**	2.36E + 7	3.96E + 7	8.41E + 5	6.18E + 5	5.09E + 5	6.01E + 6	9.15E + 6
	STD	**1.71E + 5**	9.94E + 6	1.27E + 7	9.31E + 5	4.57E + 5	4.23E + 5	2.77E + 6	3.81E + 6
F11	AVG	3.61E + 3	9.43E + 7	3.09E + 7	7.37E + 3	**2.74E + 3**	2.87E + 3	3.08E + 5	6.59E + 6
	STD	**3.43E + 3**	5.91E + 7	2.68E + 7	8.83E + 3	3.53E + 3	3.63E + 3	1.57E + 5	5.44E + 6
F12	AVG	**1.85E + 3**	6.73E + 10	7.59E + 10	3.70E + 3	2.85E + 3	3.86E + 3	2.29E + 5	2.39E + 8
	STD	**4.88E + 2**	9.12E + 10	2.10E + 11	9.27E + 2	7.44E + 2	1.60E + 3	2.54E + 5	3.24E + 8
F13	AVG	**1.56E + 3**	1.76E + 3	2.13E + 3	1.64E + 3	1.57E + 3	1.65E + 3	1.68E + 3	1.58E + 3
	STD	1.38E + 1	4.86E + 1	2.22E + 2	3.07E + 1	9.74	5.77E + 1	3.65E + 1	**6.58**
F14	AVG	2.54E + 3	4.21E + 3	3.50E + 3	4.45E + 3	2.44E + 3	**2.04E + 3**	2.25E + 3	2.76E + 3
	STD	3.79E + 2	5.58E + 2	6.95E + 2	5.84E + 2	1.93E + 2	**9.36E + 1**	1.39E + 2	1.43E + 2
F15	AVG	2.44E + 3	2.93E + 3	2.99E + 3	**2.29E + 3**	2.84E + 3	2.61E + 3	2.95E + 3	2.90E + 3
	STD	1.76E + 2	1.55E + 2	9.67E + 1	3.78E + 2	**3.09E + 1**	1.12E + 2	3.99E + 1	3.84E + 1

Significant values are given in bold.

Upon scrutinizing the statistical results of SNCPO on the CEC2015 benchmark suite, it becomes evident that SNCPO outperforms seven competing optimization algorithms in both convergence accuracy and stability. Specifically, for unimodal functions F1 and F2, SNCPO consistently attains more optimal solutions compared to other approaches, such as MFO, EDO, and GWO. This clearly demonstrates the enhanced exploitation capabilities of SNCPO, which stem from its strategic incorporation of competitive learning and adaptive salp navigation techniques. The ability of SNCPO to refine solutions efficiently allows it to surpass traditional optimization techniques, making it highly suitable for unimodal function optimization. For multimodal functions (F3–F5), SNCPO continues to demonstrate its robustness by effectively navigating complex search landscapes. Although GWO exhibits strong convergence behavior in F3, SNCPO maintains competitive performance across these functions, particularly in F4 and F5, where its AVG values surpass those of PO, AO, and SCA. This suggests that the integration of salp navigation and competitive learning strategies significantly enhances the algorithm’s ability to escape local optima and explore promising regions of the search space. The capacity to transition seamlessly between exploration and exploitation enables SNCPO to exhibit superior optimization performance in problems where conventional optimizers struggle.

While GWO achieves the best results for F3, SNCPO’s performance aligns with the No Free Lunch (NFL) theorem, which asserts that no single optimization algorithm can universally outperform all others across all problem types. Nevertheless, SNCPO continues to demonstrate strong convergence characteristics in multimodal functions, highlighting its adaptability and robustness. In the case of hybrid functions (F6–F8), which assess an algorithm’s ability to balance exploration and exploitation effectively, SNCPO exhibits notable performance improvements. Although its performance in F6 is slightly less optimal compared to other methods, it demonstrates a significant advantage in F7 and F8, achieving superior convergence results. The improved performance in these functions can be attributed to SNCPO’s adaptive exploration–exploitation mechanism, which dynamically adjusts the search strategy based on the characteristics of the optimization problem. Similarly, for composite functions (F9–F15), which present highly complex and irregular search landscapes, SNCPO exhibits remarkable performance, particularly in F10, F12, and F13. The superior performance of SNCPO in these functions underscores its capability to effectively manage optimization problems that require simultaneous exploration of multiple diverse regions while refining solutions to achieve high accuracy. This success is largely due to the combined impact of competitive learning and adaptive salp navigation strategies, which provide the algorithm with a refined ability to transition smoothly between search phases. While SNCPO generally outperforms other optimization techniques, there are instances where it achieves slightly less optimal solutions compared to MFO and EDO. However, the overall statistical results confirm its reliability and robustness, as evidenced by its consistently strong performance across multiple problem types.

**Fig. 4:**
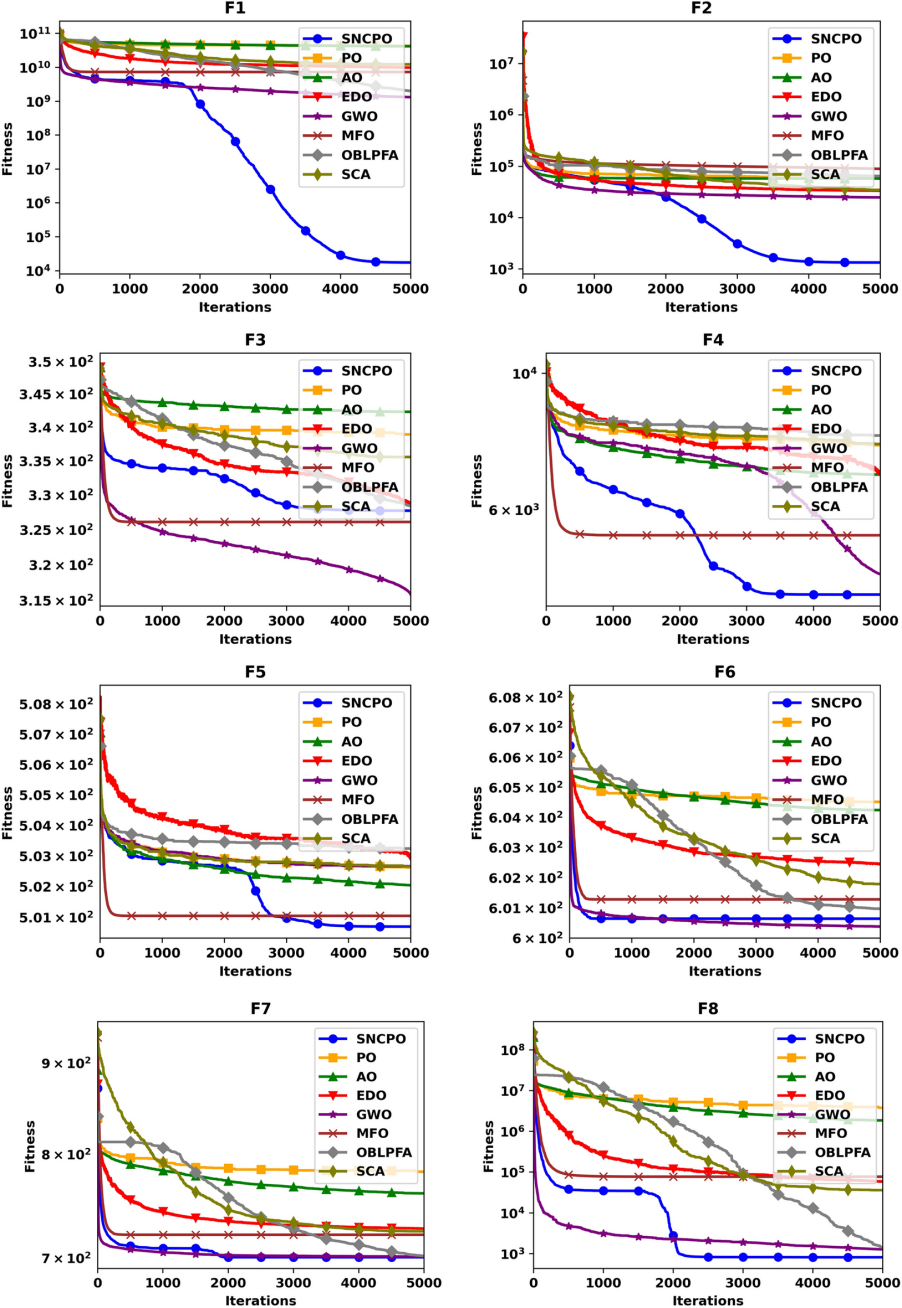

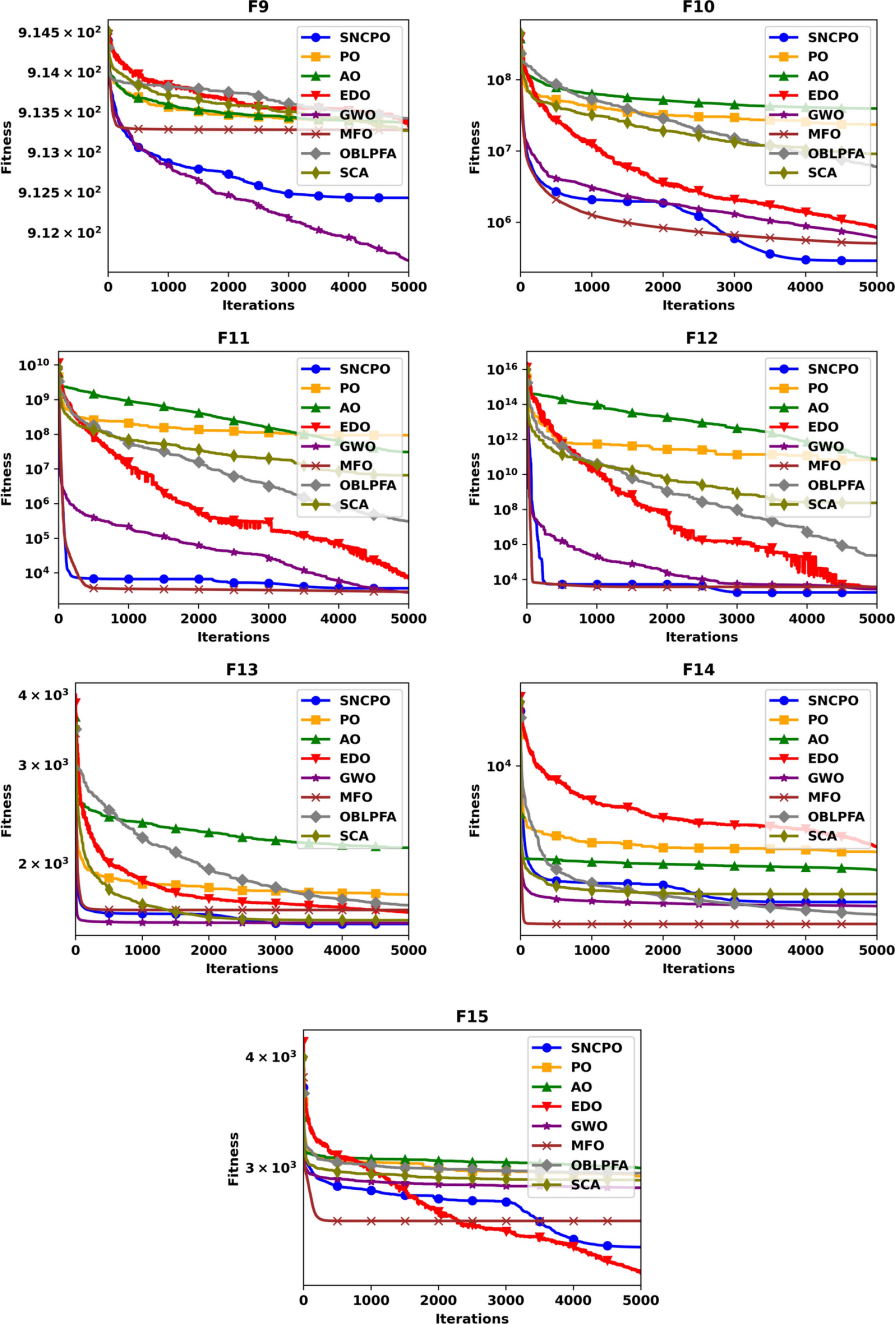
Convergence chart of SNCPO and other optimizers on CEC2015.

**Fig. 5:**
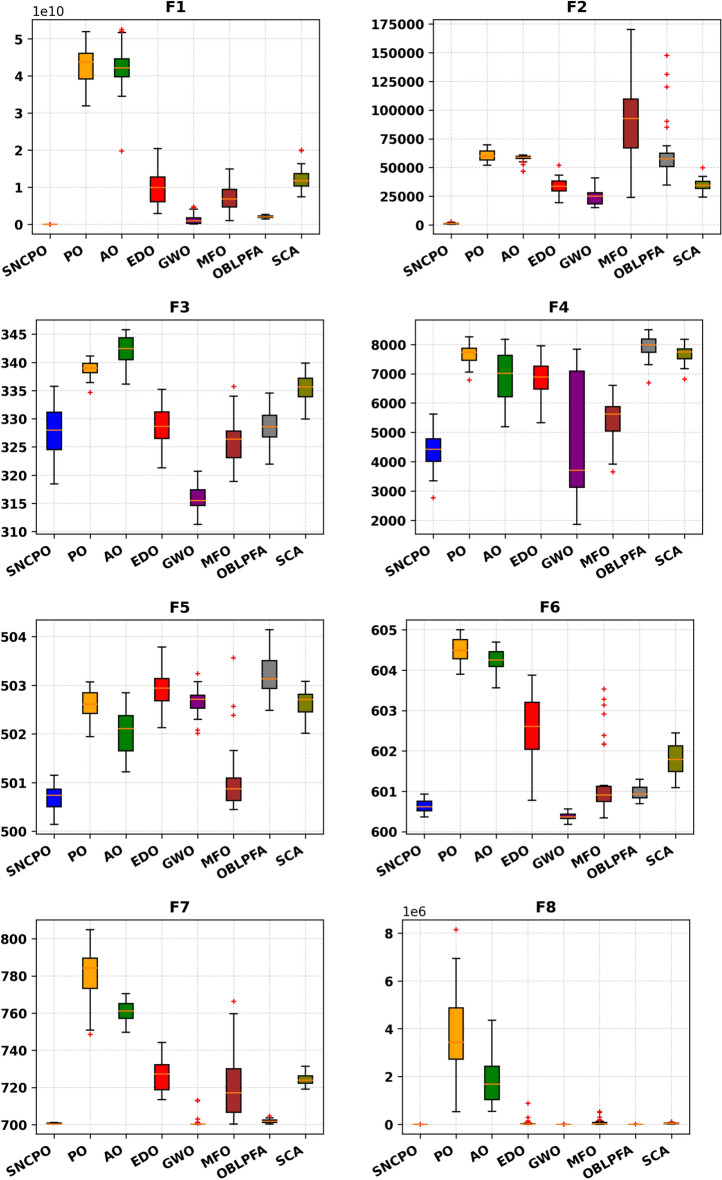

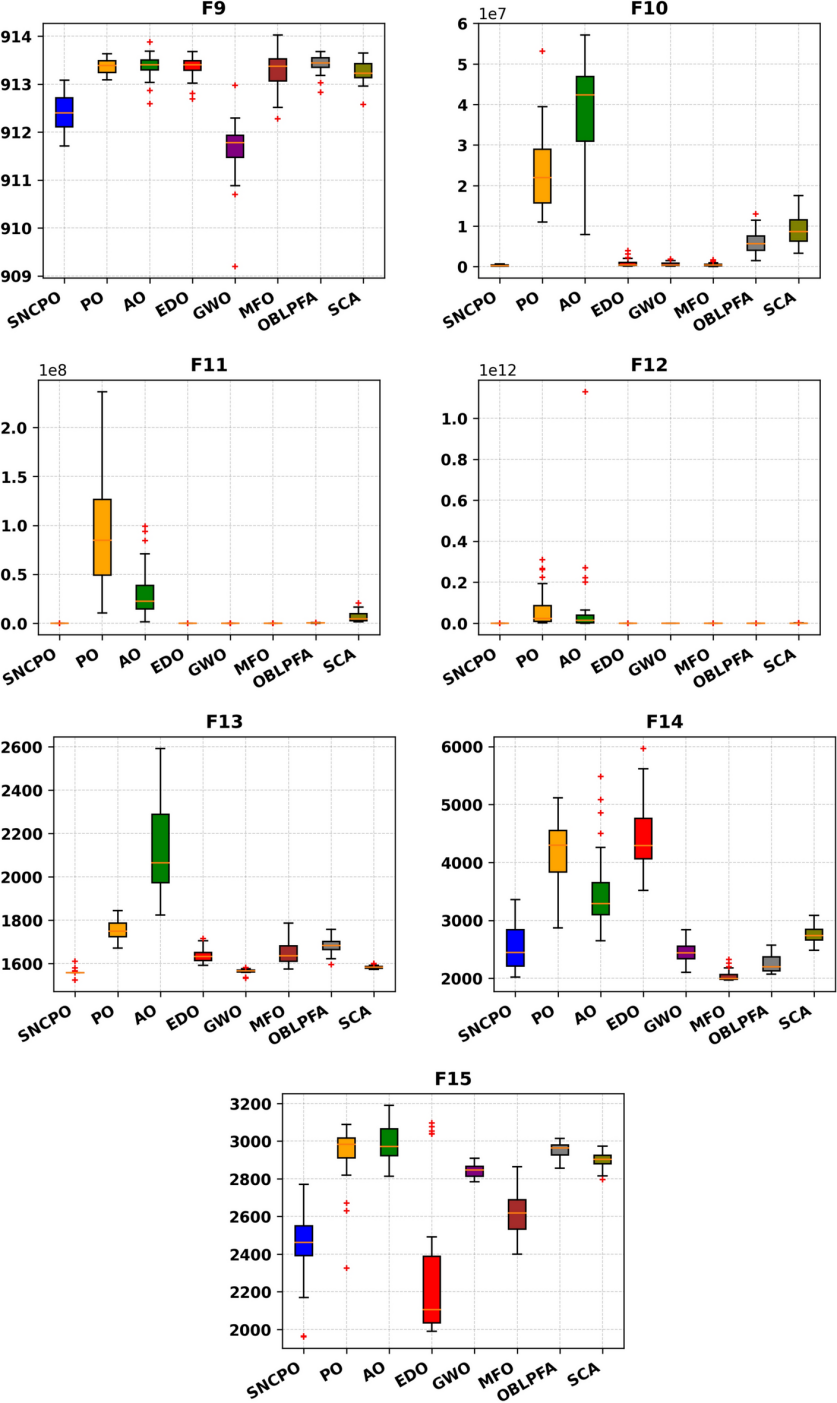
Box plots of SNCPO and other optimizers on CEC2015.

Although Table 2 provides valuable statistical insights into the numerical performance of SNCPO and its comparative algorithms, it does not offer a clear depiction of their convergence behaviors over iterations. This crucial aspect of algorithm evaluation is instead illustrated in Figs. 4 and 5, which present convergence curves and box plots, respectively. Figure 4, which showcases the convergence trajectories of SNCPO and other algorithms, reveals that SNCPO achieves significantly faster convergence rates compared to most competing optimizers across the majority of benchmark functions. This rapid convergence can be attributed to SNCPO’s salp navigation and competitive learning strategies, which enable search agents to thoroughly explore promising solution regions in the early stages of optimization while ensuring rapid convergence toward optimal solutions in later iterations. As depicted in Fig. 4, SNCPO demonstrates a gradual yet decisive descent in functions F1 and F2, highlighting its superior exploitation capabilities compared to PO, AO, EDO, GWO, OBLPFA, MFO, and SCA. This performance advantage arises from the integration of competitive learning refinement techniques and the adaptive salp navigation mechanism, both of which facilitate an effective and adaptive transition between exploration and exploitation. For multimodal functions (F3–F5), the convergence patterns further reinforce SNCPO’s superior search efficiency. While GWO exhibits noteworthy convergence characteristics in F3, SNCPO demonstrates exceptional local escaping capabilities in F4 and F5. This is evident in the continued descent of its convergence curve, in contrast to the relatively stagnant trends observed in OBLPFA, SCA, and EDO. SNCPO’s improved performance in these functions is a direct consequence of its enhanced adaptive exploration strategy, which enables it to avoid premature convergence and systematically refine solutions to achieve optimal accuracy. In hybrid functions (F6–F8), which pose a significant challenge due to their intricate mixture of multiple function landscapes, SNCPO displays a well-balanced transition between exploration and exploitation. However, while it shows slightly suboptimal performance in F6, its ability to dynamically adjust its search behavior results in superior convergence in F7 and F8. The enhanced adaptability of SNCPO in these functions ensures that it consistently identifies high-quality solutions, reinforcing its competitive advantage over conventional algorithms. Lastly, in composite functions, which represent some of the most complex optimization landscapes, SNCPO achieves outstanding performance, particularly in F10, and F12. This success can be attributed to its refined exploitation-exploration balance, which is facilitated by the synergistic effects of competitive learning and the adaptive salp navigation strategy. These methodological enhancements collectively contribute to SNCPO’s strong convergence efficiency, positioning it as a powerful tool for addressing complex real-world optimization problems.

Beyond convergence analysis, the stability and reliability of SNCPO were further assessed through box plot evaluations, as illustrated in Fig. 5. The box plots provide a statistical representation of solution variability across multiple independent runs, thereby offering insights into the consistency of each algorithm. As seen in Fig. 5, the reduced lengths of SNCPO’s box plots indicate minimal variation across different optimization runs, confirming its strong reliability and stability. This consistency is particularly important in practical applications, where reliable convergence to high-quality solutions is essential. The observed stability of SNCPO can be attributed to its robust search strategy, which prevents erratic behavior and ensures that the algorithm maintains a steady convergence trajectory.

### Statistical and convergence analysis of compared optimizers on the CEC2020

To rigorously evaluate the optimization capabilities of the proposed SNCPO algorithm, the CEC2020 benchmark functions were employed to assess its effectiveness in comparison with state-of-the-art optimization techniques. These benchmark functions, which encompass unimodal, multimodal, hybrid, and composite functions, serve as a comprehensive test to validate the efficacy and robustness of optimization algorithms in solving complex numerical optimization problems. The experimental outcomes for the SNCPO algorithm, alongside the comparative optimizers, are systematically presented in Table 3. As evidenced in Table 3, the SNCPO algorithm consistently outperforms the other algorithms across multiple benchmark functions. Specifically, for functions F16, F17, F18, F19, F20, F21, F22, F24, and F25, SNCPO achieves significantly lower average (AVG) values, demonstrating its superior convergence properties and ability to attain high-quality solutions. More notably, in the case of unimodal functions F17, F18, and F19, SNCPO exhibits remarkable performance by achieving the best mean values across all competing algorithms. This superior performance underscores SNCPO’s enhanced exploitation capabilities, which can be attributed to its refined solution refinement strategies facilitated by the integration of the competitive learning technique and the adaptive salp navigation strategy.

**Table 3:** Experimental result of SNCPO and other optimizers on CEC2020.

		SNCPO	PO	AO	EDO	GWO	MFO	OBLPFA	SCA
F16	AVG	**4.66E + 3**	4.39E + 10	3.61E + 10	1.51E + 10	1.92E + 9	9.22E + 9	3.38E + 9	1.45E + 10
	STD	**5.82E + 3**	5.92E + 9	4.88E + 9	7.43E + 9	1.64E + 9	5.34E + 9	6.74E + 8	2.27E + 9
F17	AVG	**7.16E + 5**	5.46E + 12	4.81E + 12	1.36E + 12	2.14E + 11	1.04E + 12	3.71E + 11	1.54E + 12
	STD	**8.77E + 5**	9.37E + 11	5.23E + 11	5.26E + 11	1.68E + 11	4.98E + 11	7.30E + 10	1.99E + 11
F18	AVG	**1.39E + 5**	1.66E + 12	1.35E + 12	5.03E + 11	5.69E + 10	4.62E + 11	1.12E + 11	5.23E + 11
	STD	**2.28E + 5**	2.17E + 11	2.52E + 11	1.50E + 11	3.97E + 10	2.29E + 11	2.47E + 10	7.12E + 10
F19	AVG	**1.92E + 3**	4.82E + 5	4.02E + 5	7.01E + 3	1.99E + 3	1.48E + 4	1.96E + 3	8.57E + 3
	STD	**6.93E**	2.42E + 5	1.78E + 5	8.85E + 3	2.77E + 2	2.45E + 4	1.85E + 1	5.37E + 3
F20	AVG	**2.39E + 5**	3.74E + 7	1.30E + 8	5.23E + 5	5.34E + 5	2.26E + 6	4.96E + 6	8.01E + 6
	STD	**1.32E + 5**	1.76E + 7	5.90E + 7	4.56E + 5	3.16E + 5	3.94E + 6	2.14E + 6	3.27E + 6
F21	AVG	**9.60E + 3**	4.80E + 7	1.62E + 7	2.51E + 4	2.67E + 4	5.02E + 5	9.63E + 5	3.58E + 6
	STD	**7.99E + 3**	4.02E + 7	1.56E + 7	1.03E + 4	1.73E + 4	6.75E + 5	5.39E + 5	2.57E + 6
F22	AVG	**3.10E + 5**	2.40E + 8	4.91E + 8	1.25E + 6	8.77E + 5	4.12E + 6	1.23E + 7	2.62E + 7
	STD	**2.25E + 5**	1.09E + 8	3.34E + 8	8.64E + 5	6.00E + 5	5.37E + 6	4.12E + 6	1.42E + 7
F23	AVG	2.55E + 3	3.49E + 3	4.76E + 3	2.51E + 3	**2.41E + 3**	2.48E + 3	2.47E + 3	2.59E + 3
	STD	1.16E + 2	3.37E + 2	8.23E + 2	4.00E + 1	**1.47E + 1**	5.39E + 1	1.48E + 1	2.69E + 1
F24	AVG	**2.68E + 3**	3.06E + 4	2.89E + 4	1.39E + 4	5.19E + 3	1.16E + 4	6.67E + 3	1.33E + 4
	STD	**1.44E + 2**	2.96E + 3	1.30E + 3	4.30E + 3	1.60E + 3	1.41E + 3	7.33E + 2	9.42E + 2
F25	AVG	**2.95E + 3**	5.68E + 3	5.12E + 3	3.78E + 3	3.05E + 3	3.32E + 3	3.11E + 3	3.50E + 3
	STD	**5.19E + 1**	5.87E + 2	4.34E + 2	4.45E + 2	7.59E + 1	2.69E + 2	6.32E + 1	1.25E + 2

Significant values are given in bold.

In contrast, competing algorithms such as AO, EDO, OBLPFA, and MFO exhibit suboptimal performance in addressing unimodal functions due to their inherent limitations in achieving adequate exploitation. These algorithms tend to yield suboptimal solutions due to premature convergence and insufficient refinement mechanisms, which hinder their ability to explore promising regions effectively. SNCPO, however, overcomes these challenges by leveraging its competition-based learning approach, which enhances solution refinement and strengthens its capacity to converge toward optimal solutions with greater precision. For multimodal functions spanning F20 to F22, SNCPO once again demonstrates superior optimization capabilities, achieving the best mean values across these benchmark functions. The inherent difficulty of multimodal functions lies in their complex landscape, which consists of numerous local optima that can mislead optimization algorithms into suboptimal regions. Despite this challenge, SNCPO exhibits a strong ability to navigate these complex search spaces effectively. In contrast, EDO, GWO, MFO, and OBLPFA achieve comparatively lower mean values when solving multimodal functions, indicating their limitations in escaping local optima and maintaining solution diversity. SNCPO’s superior performance in these functions is largely attributed to its adaptive salp navigation strategy, which facilitates a balanced transition between exploration and exploitation. This adaptive mechanism ensures that the algorithm does not stagnate in local optima, thereby improving the overall convergence behavior. With regard to hybrid functions (F23–F25), SNCPO further distinguishes itself by demonstrating superior convergence capabilities, particularly in functions F24 and F25, where it achieves the best mean values. The hybrid functions in CEC2020 are designed to assess an optimizer’s ability to effectively transition between exploration and exploitation, given their composition of multiple distinct sub-functions with varying characteristics. While GWO exhibits competitive performance in handling hybrid functions and achieves a superior solution in F23, SNCPO remains the dominant algorithm in F24 and F25 due to its efficient transition strategy between exploration and exploitation. This advantage is directly linked to the fusion of the competitive learning technique with the adaptive salp navigation strategy, which collectively enhances SNCPO’s capability to dynamically adjust its search behavior based on the problem landscape.

**Fig. 6:**
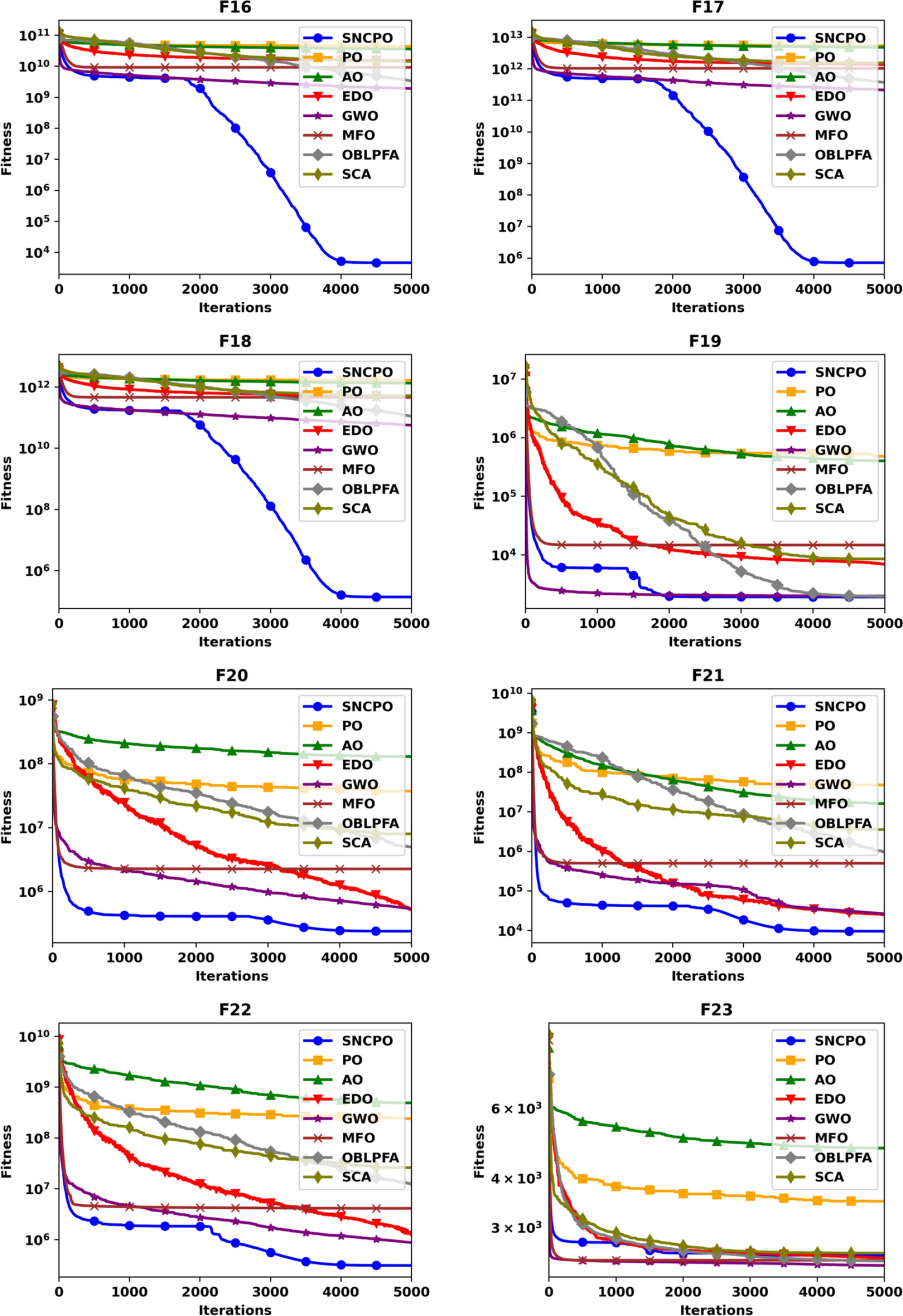

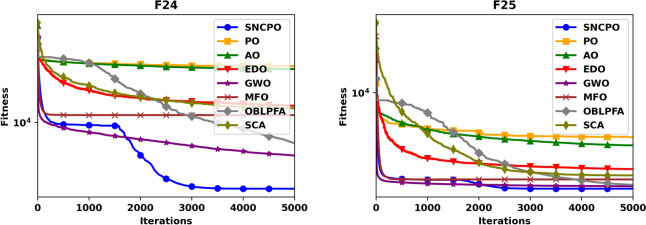
Convergence chart of SNCPO and other optimizers on CEC2020.

**Fig. 7:**
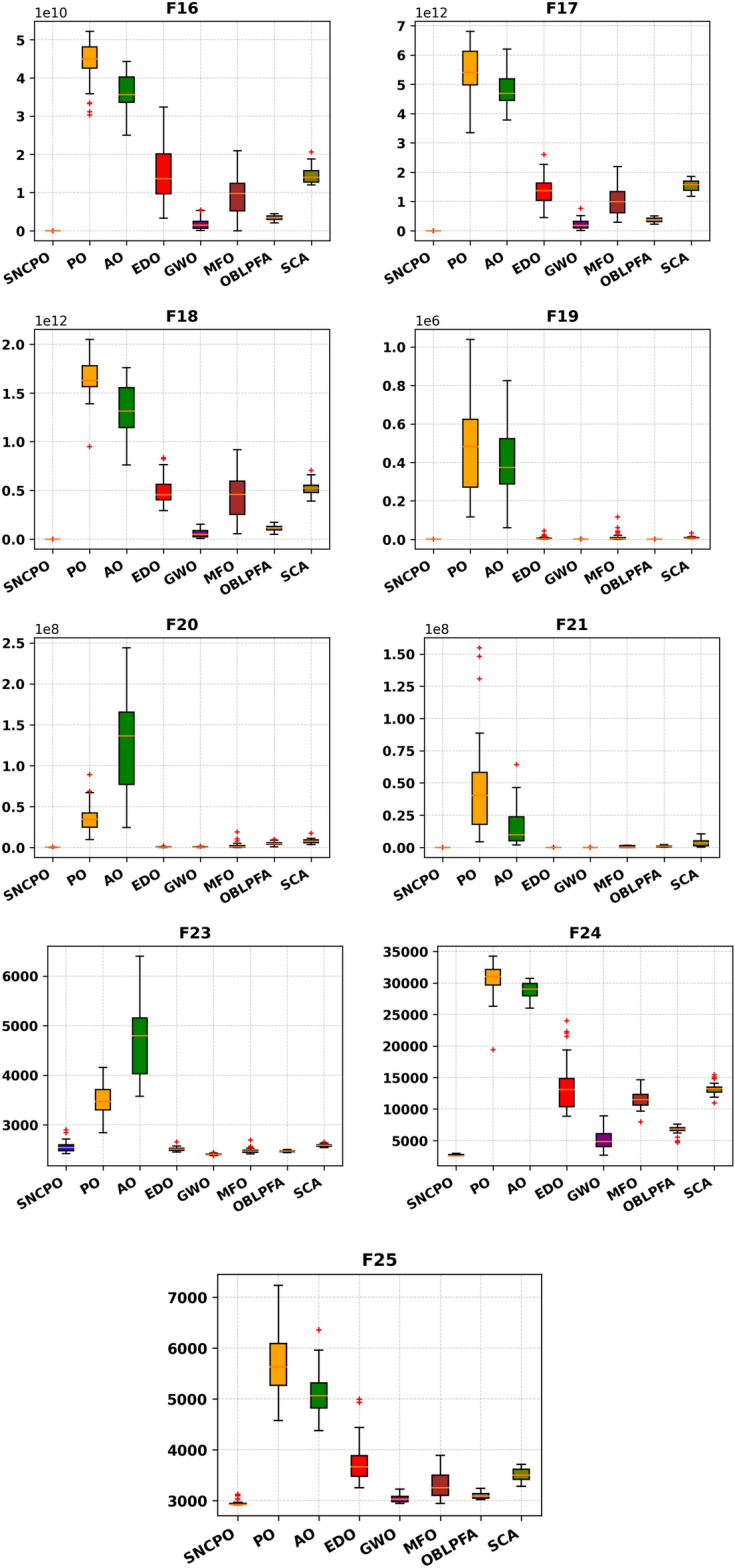
Convergence chart of SNCPO and other optimizers on CEC2020.

To further investigate the search efficiency of SNCPO and its comparative algorithms, a detailed visual examination was conducted through the analysis of convergence curves, as illustrated in Fig. 6. These convergence curves provide insights into each algorithm’s search efficiency and optimization dynamics when tackling the challenging CEC2020 benchmark test functions. By analyzing these curves, a deeper understanding of the algorithms’ convergence behaviors can be obtained, shedding light on their ability to effectively explore and exploit the search space. Notably, the proposed SNCPO algorithm demonstrates highly competitive convergence speeds, successfully identifying promising solution regions of the benchmark functions at various stages of the optimization process. A consistent pattern of a steep decline is evident across all convergence curves of SNCPO, particularly in functions F16, F17, F18, F19, F20, F21, F22, F24, and F25. This observation highlights SNCPO’s exceptional ability to rapidly identify high-quality solutions during the early stages of the optimization process. Such an ability is crucial for real-world optimization applications, where rapid convergence to optimal or near-optimal solutions is often required. Additionally, Fig. 6 provides empirical evidence of SNCPO’s robust exploration capability, as observed through the consistent spread of solutions across the optimization process. The ability to maintain exploration throughout the search space is a critical attribute of an effective optimization algorithm, ensuring that it does not become trapped in local optima. This is primarily attributed to the adaptive salp navigation strategy, which allows SNCPO to sustain a dynamic balance between exploration and exploitation. Unlike other algorithms that exhibit premature convergence or stagnation, SNCPO effectively mitigates these issues by strategically switching between search phases, thereby enhancing its overall optimization performance. Furthermore, the convergence curves reveal that SNCPO achieves a steady decline in mean values during the early iterations while maintaining variability, which signifies its capacity to explore diverse regions of the search space before converging towards the global optimum in later iterations. This gradual yet directed convergence process is instrumental in ensuring the robustness and reliability of the algorithm across multiple optimization scenarios. Beyond convergence analysis, the robustness and consistency of the SNCPO algorithm were further examined through box plot analysis, as presented in Fig. 7. Box plots offer a visual representation of the performance stability of each optimization algorithm across multiple independent runs. The results depicted in Fig. 7 highlight SNCPO’s remarkable consistency, as indicated by the reduced length of the box plots. The shorter box lengths suggest lower variability in the obtained solutions, reflecting the algorithm’s ability to consistently achieve stable and reliable optimization outcomes across different trials.

### Time complexity analysis

Tables 4 and 5 present the average computation times (in seconds) of the proposed SNCPO algorithm and traditional optimizers on the CEC2015 and CEC2020 benchmark functions. The results reveal a clear computational advantage for the AO and SNCPO algorithms over conventional methods. Notably, traditional optimizers such as GWO, MFO, OBLPFA, and SCA consistently exhibit higher computational demands, with MFO demonstrating the most pronounced inefficiency across both benchmark suites. This underperformance of MFO is attributed to its reliance on complex flame attraction mechanisms and iterative parameter adjustments, which amplify computational overhead, particularly in high-dimensional or multimodal landscapes. The AO algorithm, however, achieves the lowest average computation time across all test functions in both CEC2015 and CEC2020. This efficiency is primarily due to its minimalist design, characterized by a low-parameter configuration and reduced number of iterative loops. The absence of redundant calculations enables AO to minimize computational burden while maintaining robust search capabilities.

**Table 4:** Computation time analysis of SNCPO and other optimizers on CEC2015.

	SNCPO	PO	AO	EDO	GWO	MFO	OBLPFA	SCA
F1	18.16	7.83	**5.24**	60.61	78.78	107.95	80.21	68.54
F2	18.21	7.75	**5.21**	60.65	78.3	108.35	80.03	68.7
F3	70.82	114.01	**58.66**	114	132.12	196.33	185.64	121.87
F4	32.84	37.28	**19.83**	75.85	93.44	132.91	109.51	84.08
F5	874.43	1647.77	**861.02**	905.89	924.97	1438.1	1634.55	912.65
F6	19.45	8.55	**6.5**	61.26	79.67	92.47	69.09	69.56
F7	19.51	8.63	**6.5**	61.31	78.51	92.43	61.26	69.63
F8	121.16	176.37	**108.21**	159.26	165.97	182.88	171.77	161.84
F9	72.61	97.23	**59.88**	104.35	113.2	110.74	103.83	111.18
F10	36.07	36.32	**23.17**	70.24	80.67	75.6	60.54	72.59
F11	54.81	63.44	**42.2**	81.21	96.76	84.27	75.58	88.09
F12	145.86	162.68	**134.43**	157.31	173.76	157.57	160.47	164.86
F13	34.5	26.4	**25.72**	67.74	82.68	60.53	50.95	74.56
F14	39.7	32.15	**30.7**	73.04	71.83	63.41	54.71	79.35
F15	94.97	98.55	**90.15**	122.9	101.34	104.13	103.88	111.65

Significant values are given in bold.

**Table 5:** Computation time analysis of SNCPO and other optimizers on CEC2020.

	SNCPO	PO	AO	EDO	GWO	MFO	OBLPFA	SCA
F16	17.61	7.74	**5.08**	58.68	77.56	106.04	78.51	67.13
F17	18.16	8.71	**5.51**	60.36	78.89	108.09	80.56	68.22
F18	18.39	8.72	**5.51**	60.34	78.98	107.45	80.68	68.29
F19	120.22	214.25	**105.62**	159.05	173.07	241.99	258.75	165.27
F20	36.35	44.58	**23.35**	72.65	83.7	105.38	95.16	78.86
F21	48.89	66.69	**35.84**	77.79	91.18	115.69	106.68	83.35
F7	45.83	58.61	**32.68**	73.57	80.61	76.26	94.71	77.26
F8	49.84	68.87	**40.62**	73.68	86.54	68.02	71.3	78.86
F9	40.73	51.07	**32.04**	65.06	65.99	61.15	53.59	71.44
F10	37.9	45.57	**28.52**	62.25	45.93	57.49	50.85	56.42

Significant values are given in bold.

When comparing the SNCPO algorithm to its predecessor, the traditional PO algorithm. As shown in Table 4 (CEC2015), the original PO algorithm requires significantly longer computation times than SNCPO in the majority of functions, with exceptions observed only in F1, F2, F6, and F7. A similar pattern is evident in Table 5 (CEC2020), where SNCPO outperforms PO in most functions except F16, F17, and F18. This disparity is rooted in the redesign of SNCPO optimization process, which eliminates redundant mathematical operations inherent in the traditional PO framework. Specifically, SNCPO adopts a dual-update strategy: winners are refined using the SSA operator, while losers are updated via PO’s communication strategy. This structural simplification reduces computational loops and arithmetic complexity, thereby minimizing overhead without compromising solution quality. While SNCPO demonstrates superior average performance, the residual cases where PO outperforms SNCPO highlight the influence of problem-specific characteristics. For instance, in low-dimensional or unimodal functions (e.g., F1, F2), the overhead of SNCPO’s SSA operator may outweigh its benefits, whereas PO’s straightforward position update mechanism executes more efficiently. Also, functions with regular or symmetric landscapes align more closely with PO’s inherent design principles, enabling it to converge rapidly with less accurate results. Conclusively SNCPO showed improved computational cost compared PO.

### Non-parametric analysis

In this study, the Wilcoxon signed-rank test^61^ was employed as a nonparametric statistical method with a significance threshold of 0.05. According to the Wilcoxon signed-rank test, if the p-value falls below the significance level, it implies a statistically significant improvement. On the contrary, if the p-value surpasses the significance level, it implies no notable difference between the methods being compared. This approach allows for a robust performance contrast between the SNCPO and compared algorithms. Additionally, the Friedman test^62^ was utilized to assess the average rankings of the algorithms, providing additional insight into their overall performance. As shown in Table 6, the results of the Friedman test reveal that the SNCPO ranks first among the compared algorithms, demonstrating its ability to deliver superior solutions on the benchmark test suite. A detailed examination of the Wilcoxon signed-rank test results highlights the strong competitive edge of the SNCPO over other optimizers, with statistically significant improvements observed in the majority of instances. However, it is important to acknowledge that GWO exhibited enhanced performance in a few benchmark functions. Despite this, the SNCPO consistently outperforms the majority of its counterparts, reaffirming its effectiveness and reliability in optimization scenarios.

**Table 6:** Non-parametric test of SNCPO and other optimizers.

		SNCPO	PO	AO	EDO	GWO	MFO	OBLPFA	SCA
CEC2015	Wilcoxon P-Value	–	6.550E−04	6.533E−04	2.600E−03	6.403E−02	3.305E−02	4.637E−03	6.533E−04
	Friedman Mean Value	**1.8**	6.97	6.57	4.7	2.27	3.6	4.8	5.3
	Friedman Rank	**1**	8	7	4	2	3	5	6
CEC2020	Wilcoxon P-Value	–	5.062E−03	5.062E−03	6.910E−03	1.252E−02	6.910E−03	9.344E−03	5.062E−03
	Friedman Mean Value	**1.40**	7.70	7.30	4.30	2.20	4.10	3.40	5.60
	Friedman Rank	**1**	8	7	5	2	4	3	6

Significant values are given in bold.

### Exploration vs exploitation

**Fig. 8:**
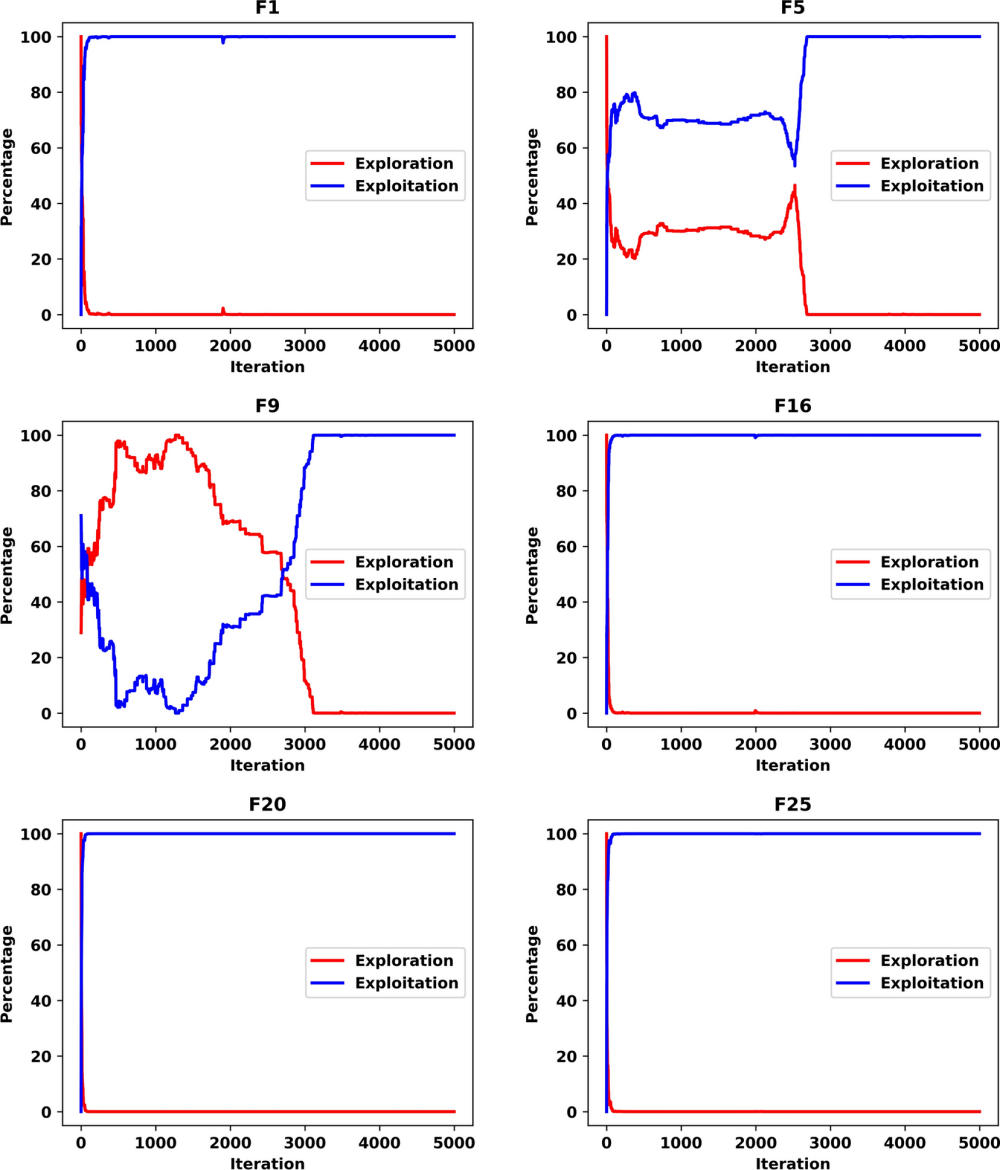
Exploration vs exploitation analysis of SNCPO.

Analyzing the equilibrium between the exploration and exploitation stages of the SNCPO can yield valuable insights for addressing real-world optimization challenges. To ensure a thorough evaluation of the SNCPO, this study delves into the algorithm’s exploration and exploitation abilities based on the benchmark functions examined. Figure 8 illustrates the balance between the exploratory and exploitative behaviors of the SNCPO. In the figure, the red curve represents the algorithm’s exploration tendencies, and the blue curve highlights its exploitation abilities. From Fig. 8, it is evident that for relatively straightforward unimodal functions (F1, F16) and simple multimodal functions (F5), the exploration ratio remains low meaning SNCPO was swift in identifying promising regions quickly. In the case of F5, the SNCPO briefly alternates between exploration and exploitation during the early stages of iteration, aiming to maintain a dynamic equilibrium. As observed in Fig. 8, when tackling more intricate hybrid functions (F20) and composition functions (F9, F25), the SNCPO sustains a balanced interplay between exploration and exploitation. However, there is a noticeable decline in exploration as the algorithm identifies promising regions, followed by an intensified focus on exploitation.

An exception is observed in F9, where the SNCPO initially increases the proportion of exploration while reducing exploitation until the mid-iteration phase. This adjustment serves to expand the global search, thereby mitigating the risk of the algorithm being confined in deceptive local optima when handling more complex functions. The SNCPO enhances its exploration behavior through the salp navigation and PO mechanism, which introduces diversity into the population. By continuously adapting the search domain in this manner, the algorithm ultimately converges to the global optimum as the slap navigation transitions to the exploitation strategy.

### Engineering problem

#### Welded beam problem (WBP)

**Fig. 9:**
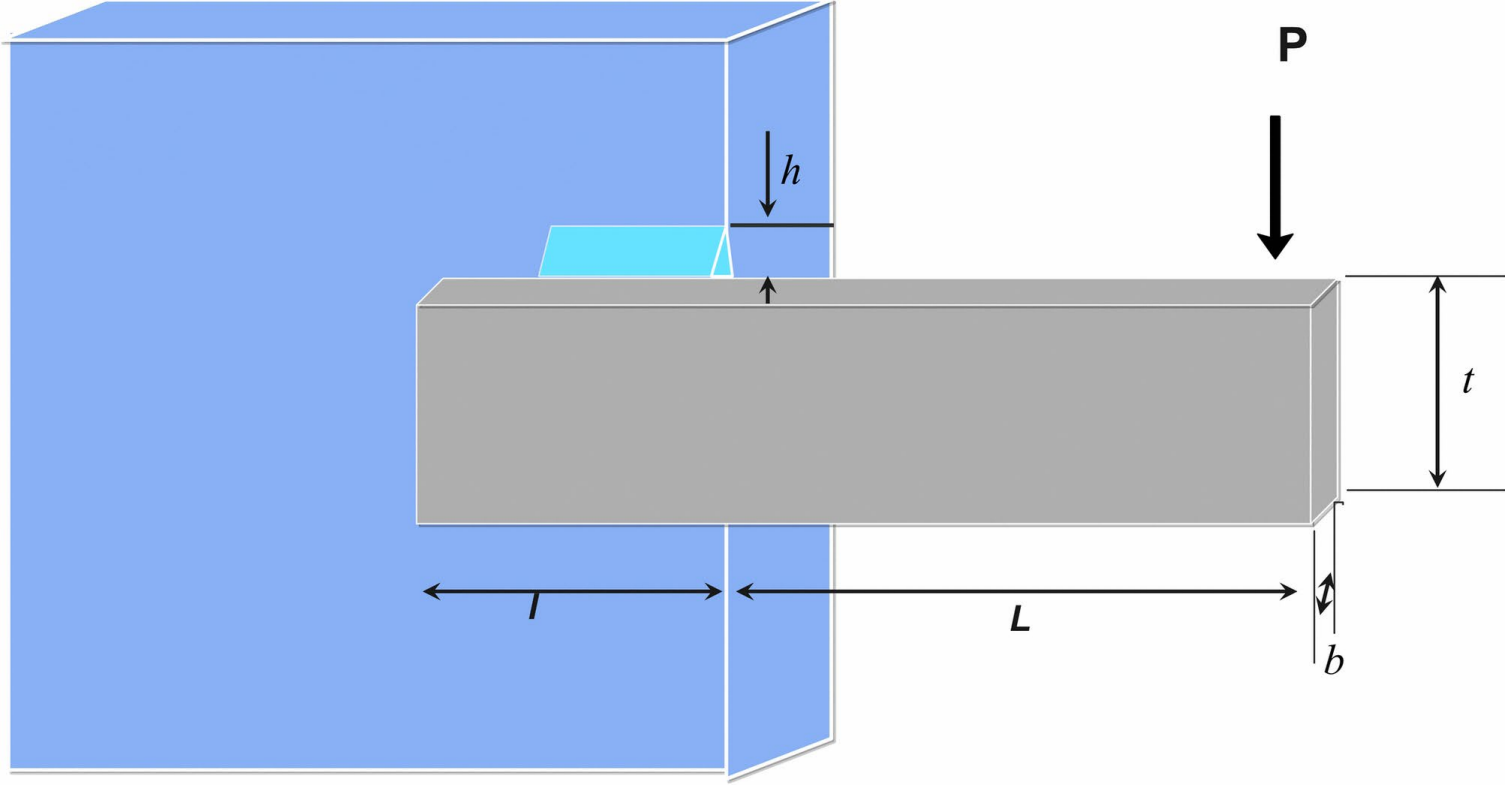
Welded beam design illustration.

The Welded Beam Design (WBD) is a real-world engineering problem focused on minimizing the fabrication cost of a welded beam. Figure 9 illustrates the design configuration along with its corresponding mathematical formulation^63^. Equation (24) and (25) are minimized based on the constraints in Eq. (26) to (40).

24 $$X=\left[{x}_{1},{x}_{2},{x}_{3},{x}_{4}\right]=[h,l,t,b]$$

25 $$f(x)=1.10471{x}_{1}^{2}{x}_{2}+0.04811{x}_{3}{x}_{4}\left(14.0+{x}_{2}\right)$$

26 $${g}_{1}(x)=\tau (x)-\text{13,600}\le 0$$

27 $${g}_{2}(x)=\sigma (x)-\text{30,000}\le 0$$

28 $${g}_{3}(x)={x}_{1}-{x}_{4}\le 0$$

29 $${g}_{4}(x)=0.10471{x}_{1}^{2}+0.04811{x}_{3}{x}_{4}\left(14+{x}_{2}\right)-5.0\le 0$$

30 $${g}_{5}(x)=0.125-{x}_{1}\le 0$$

31 $$g_{6} \left( x \right) = \delta \left( x \right) - 0.25 \le 0$$

32 $$g_{7} \left( x \right) = 6000 - p_{c} \left( x \right) \le 0$$

33 $$\tau \left( x \right) = \sqrt {\left( {\tau{\prime} } \right)^{2} + \left( {2\tau \tau{\prime} } \right)\frac{{x_{2} }}{2R} + \left( {\tau^{^{\prime\prime}} } \right)^{2} }$$

34 $$\tau^{\prime} = \frac{6000}{{\sqrt 2 x_{1} x_{2} }},\tau^{\prime\prime} = \frac{MR}{J}$$

35 $$M=6000\left(14+\frac{{x}_{2}}{2}\right)$$

36 $$R=\sqrt{\frac{{x}_{2}^{2}}{4}+{\left(\frac{{x}_{1}+{x}_{3}}{2}\right)}^{2}}$$

37 $$J=2\left\{{x}_{1}{x}_{2}\sqrt{2}\left[\frac{{x}_{2}^{2}}{12}+{\left(\frac{{x}_{1}+{x}_{3}}{2}\right)}^{2}\right]\right\}$$

38 $$\sigma (x)=\frac{\text{504,000}}{{x}_{4}{x}_{3}^{2}}$$

39 $$\delta (x)=\frac{\text{65,856,000}}{\left(30\cdot {10}^{6}\right){x}_{4}{x}_{3}^{3}}$$

40 $${p}_{c}(x)=\frac{4.013\left(30\cdot {10}^{6}\right)\sqrt{\frac{{x}_{3}^{2}{x}_{4}^{6}}{36}}}{196}\left(1-\frac{{x}_{3}}{28}\sqrt{\frac{30\cdot {10}^{6}}{4\left(12\cdot {10}^{6}\right)}}\right)$$

While: $$0.1\le {x}_{1},{x}_{4}\le 2\text{ and }0.1\le {x}_{2},{x}_{3}\le 10$$.

#### Compression spring problem (CSP)

**Fig. 10:**
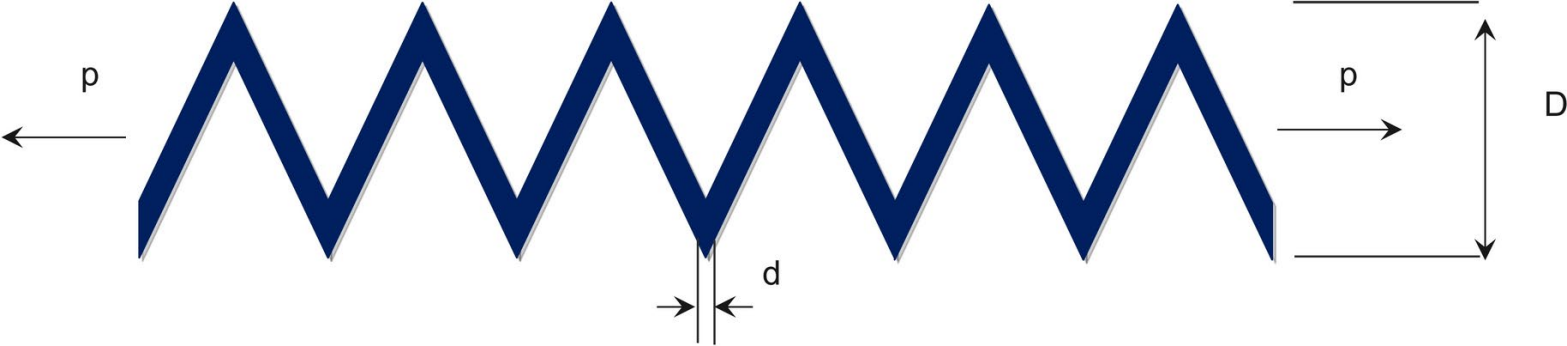
Tension/compression spring design illustration.

The objective of the CSP is to reduce the spring’s weight, posing a practical challenge in engineering. Figure 10 illustrates the schematic of the design, and the mathematical model for it is presented below ^63^. Equation (41) and (42) are minimized based on the constraints in Eq. (43) to (46).

41 $$X=\left[{x}_{1},{x}_{2},{x}_{3}\right]=[d,D,P]$$

42 $$f(x)=\left({x}_{3}+2\right){x}_{2}{x}_{1}^{2}$$

43 $${g}_{1}(x)=1-\frac{{x}_{2}^{3}{x}_{3}}{\text{71,785}{x}_{1}^{4}}\le 0$$

44 $${g}_{2}(x)=\frac{4{x}_{2}^{2}-{x}_{1}{x}_{2}}{\text{12,566}\left({x}_{2}{x}_{1}^{3}\right)}+\frac{1}{5108{x}_{1}^{2}}-1\le 0$$

45 $${g}_{3}(x)=1-\frac{140.45{x}_{1}}{{x}_{2}^{2}{x}_{3}}\le 0$$

46 $${g}_{4}(x)=\frac{{x}_{1}+{x}_{2}}{1.5}-1\le 0$$

$$0.05 \le x_{1} \le 2,{ }0.25 \le x_{2} \le 1.3{\text{ and }}2 \le x_{3} \le 15.$$

#### Speed reducer design (SRD)

**Fig. 11:**
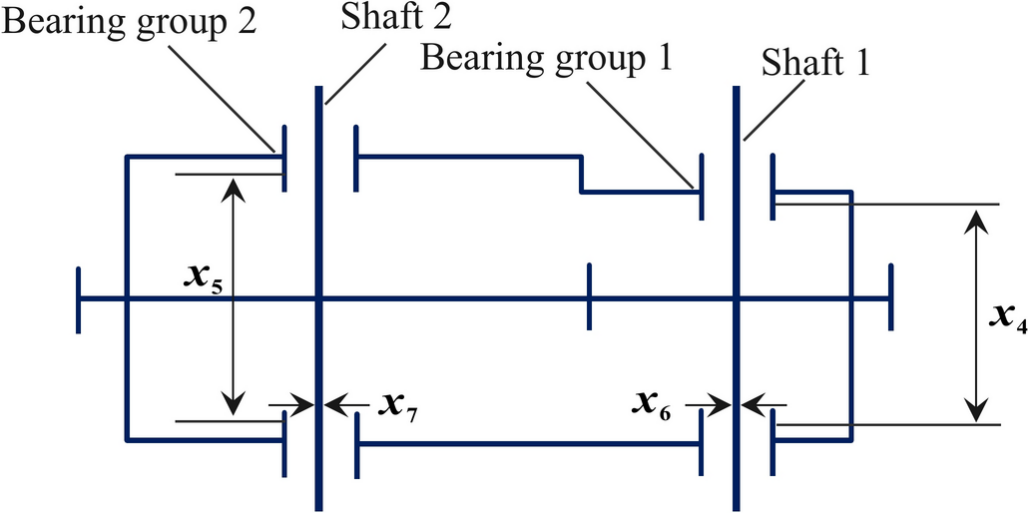
Speed reducer design.

The optimization of speed reducer designs has attracted significant interest within the field of mechanical engineering, given its crucial role in controlling machine speeds. The main goal of this optimization process is to minimize the weight of the speed reducer while adhering to the constraints set by its essential components. Seven key design variables are critical in determining the weight: the gear face width (${x}_{1}$), the teeth module (${x}_{2}$), the number of pinion teeth (${x}_{3}$), the length of the first shaft between bearings (${x}_{4}$), the length of the second shaft between bearings (${x}_{5}$), and the diameters of the first (${x}_{6}$) and second shafts (${x}_{7}$)^64^. A schematic representation of this problem is provided in Fig. 11, and the mathematical formulation is expressed in Eq. (47) subject to Eq. (48) to (58).

47 $$\begin{array}{cc}f(x)=& 0.7854{x}_{1}{x}_{2}^{2}\left(3.3333{x}_{3}^{2}+14.9334{x}_{3}-43.0934\right)\\ & -1.508{x}_{1}\left({x}_{6}^{2}+{x}_{7}^{2}\right)+7.4777\left({x}_{6}^{3}+{x}_{7}^{3}\right)\\ & +0.7854\left({x}_{4}{x}_{6}^{2}+{x}_{5}{x}_{7}^{2}\right)\end{array}$$

48 $${g}_{1}(x)=\left(27/{x}_{1}{x}_{2}^{2}{x}_{3}\right)-1\le 0$$

49 $${g}_{2}(x)=\left(397.5/{x}_{1}{x}_{2}^{2}{x}_{3}\right)-1\le 0$$

50 $${g}_{3}(x)=\left(1.93{x}_{4}^{3}/{x}_{2}{x}_{3}{x}_{6}^{4}\right)-1\le 0$$

51 $${g}_{4}(x)=\left(1.93{x}_{5}^{3}/{x}_{2}{x}_{3}{x}_{7}^{4}\right)-1\le 0$$

52 $${g}_{5}(x)=\left(1/110{x}_{6}^{3}\right)\sqrt{{\left(745{x}_{4}/{x}_{2}{x}_{3}\right)}^{2}+16.9\times {10}^{6}}-1\le 0$$

53 $${g}_{6}(x)=\left(1/85{x}_{7}^{3}\right)\sqrt{{\left(745{x}_{4}/{x}_{2}{x}_{3}\right)}^{2}+157.5\times {10}^{6}}-1\le 0$$

54 $${g}_{7}(x)=\left({x}_{2}{x}_{3}/40\right)-1\le 0$$

55 $${g}_{8}(x)=\left(5{x}_{2}^{2}/{x}_{1}\right)-1\le 0$$

56 $${g}_{9}(x)=\left({x}_{1}/12{x}_{2}\right)-1\le 0$$

57 $${g}_{10}(x)=\left(\left(1.5{x}_{6}+1.9\right)/{x}_{4}\right)-1\le 0$$

58 $${g}_{11}(x)=\left(\left(1.1{x}_{7}+1.9\right)/{x}_{5}\right)-1\le 0$$

with $$\begin{gathered} 2.6 \le x_{1} \le 3.6,{\text{~}}0.7 \le x_{2} \le 0.8,{\text{~}}2.6 \le x_{1} \le 3.6,{\text{~}}17 \le x_{3} \le 28,{\text{~}}7.3 \hfill \\ \le x_{4} \le 7.8,\;7.8 \le x_{5} \le 8.3,{\text{~}}2.9 \le x_{6} \le 3.9\;{\text{and }}5 \le x_{7} \le 5.5\;. \hfill \\ \end{gathered}$$

#### Three bar Truss design (TBTD)

**Fig. 12:**
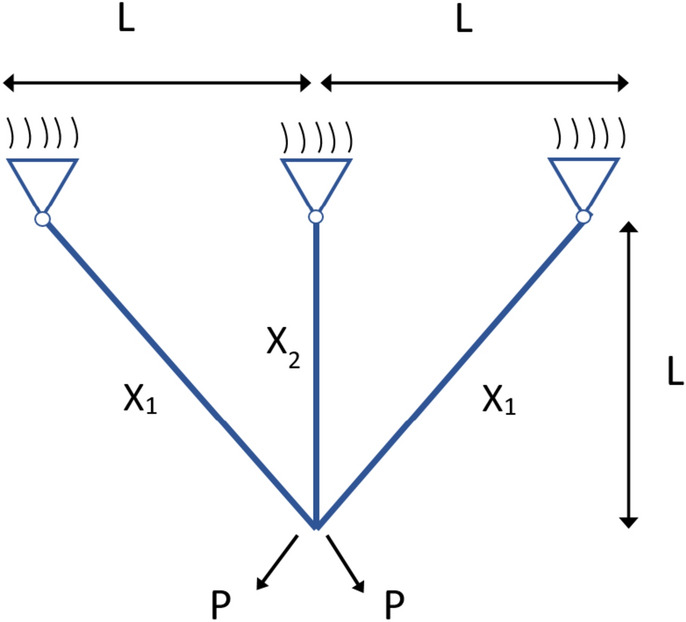
Three-bar Truss design parameters.

The aim of this problem is to reduce the weight of the structure, as depicted in Fig. 12^65^. This optimization task can be redefined as a problem involving two parameters, denoted as ${x}_{1}$ and ${x}_{2}$. Let $X$ = [${x}_{1}$,${x}_{2}$], where ${x}_{1}$ and ${x}_{2}$ are randomly chosen from the interval [0, 1]. The mathematical representation of Fig. 12 is provided in Eq. (59), while the constraints are specified in Eqs. (60), (61), and (62).

59 $$f\left( {x_{1} ,x_{2} } \right) = l \times \left( {2\sqrt 2 x_{1} + x_{2} } \right)$$

60 $$G_{1} = \frac{{\sqrt 2 x_{1} + x_{2} }}{{\sqrt 2 x_{1} 2 + 2x_{1} x_{2} }}P - \sigma \le 0$$

61 $$G_{2} = \frac{{x_{2} }}{{\sqrt 2 x_{1} 2 + 2x_{1} x_{2} }}P - \sigma \le 0$$

62 $$G_{3} = \frac{1}{{\sqrt 2 x_{2} + x_{1} }}P - \sigma \le 0$$

where: $$l = 100{\text{cm}};P = \frac{2kN}{{{\text{cm}}^{2} }};\sigma = \frac{{2{\text{kN}}}}{{{\text{cm}}^{2} }},\;{\text{Interval}}:0 \le x_{1} ,x_{2} \le 1$$

Table 7 provides a comprehensive statistical evaluation of the experimental results obtained from the comparative analysis of SNCPO and other state-of-the-art optimization algorithms across four challenging engineering optimization problems. The experimental setup for this evaluation is consistent with previous benchmarks, with an iteration count of 5000 and a population size of 30. To ensure a fair and unbiased comparison, the parameter settings outlined in Table 1 were maintained across all competing optimization techniques. The results in Table 7 present the AVG and STD values obtained over 30 independent runs, offering valuable insights into the performance, stability, and reliability of each algorithm. A thorough assessment of the statistical findings clearly demonstrates that SNCPO emerges as a highly competitive optimization approach, consistently identifying near-optimal or optimal solutions in the majority of the tested engineering problems. Its effectiveness is highlighted by its ability to achieve the lowest AVG function values for several problem instances while maintaining competitive performance in others. These findings underscore the robustness and adaptability of SNCPO, reinforcing its potential for solving complex real-world engineering optimization tasks. More specifically, the results in Table 7 reveal that SNCPO achieved the best mean value for both the WBP and the CSP. This superior performance underscores the algorithm’s capacity to navigate complex constraint landscapes while effectively balancing exploration and exploitation. The ability of SNCPO to outperform other algorithms in these engineering problems suggests that the newly introduced search strategy effectively guides the optimization process toward optimal solutions while maintaining solution feasibility and constraint satisfaction.

**Table 7:** Engineering problem result.

		**SNCPO**	**PO**	**AO**	**EDO**	**GWO**	**MFO**	**OBLPFA**	**SCA**
WBP	AVG	**1.6833E + 00**	2.5227E + 00	1.6835E + 00	1.7133E + 00	1.6924E + 00	1.7772E + 00	1.6983E + 00	1.7908E + 00
	STD	**6.3122E−04**	4.1414E−01	1.0031E−03	1.6848E−02	2.2047E−02	1.8864E−01	2.1229E−02	1.8375E−02
CSP	AVG	**6.0761E−03**	6.0787E−03	6.0771E−03	6.1439E−03	6.0762E−03	**6.0761E−03**	**6.0761E−03**	6.0773E−03
	STD	9.1682E−09	2.2161E−06	6.6956E−07	4.4347E−05	2.2440E−08	**0.0000E + 00**	1.5594E−10	1.6897E−06
SRD	AVG	3.0001E + 03	3.1328E + 03	2.9941E + 03	3.0263E + 03	2.9931E + 03	**2.9904E + 03**	2.9968E + 03	3.0749E + 03
	STD	2.3008E + 01	4.1869E + 01	3.6154E + 00	1.8555E + 01	**3.3360E + 00**	1.0025E + 01	4.3867E + 00	2.4591E + 01
TBTD	AVG	**2.6390E + 02**	2.6416E + 02	**2.6390E + 02**	**2.6390E + 02**	2.6453E + 02	2.6394E + 02	**2.6390E + 02**	2.7025E + 02
	STD	**3.8579E−06**	1.6939E−01	1.1471E−03	1.0114E−03	3.4591E + 00	7.2278E−02	2.4909E−04	9.0563E + 00

Significant values are given in bold.

**Fig. 13:**
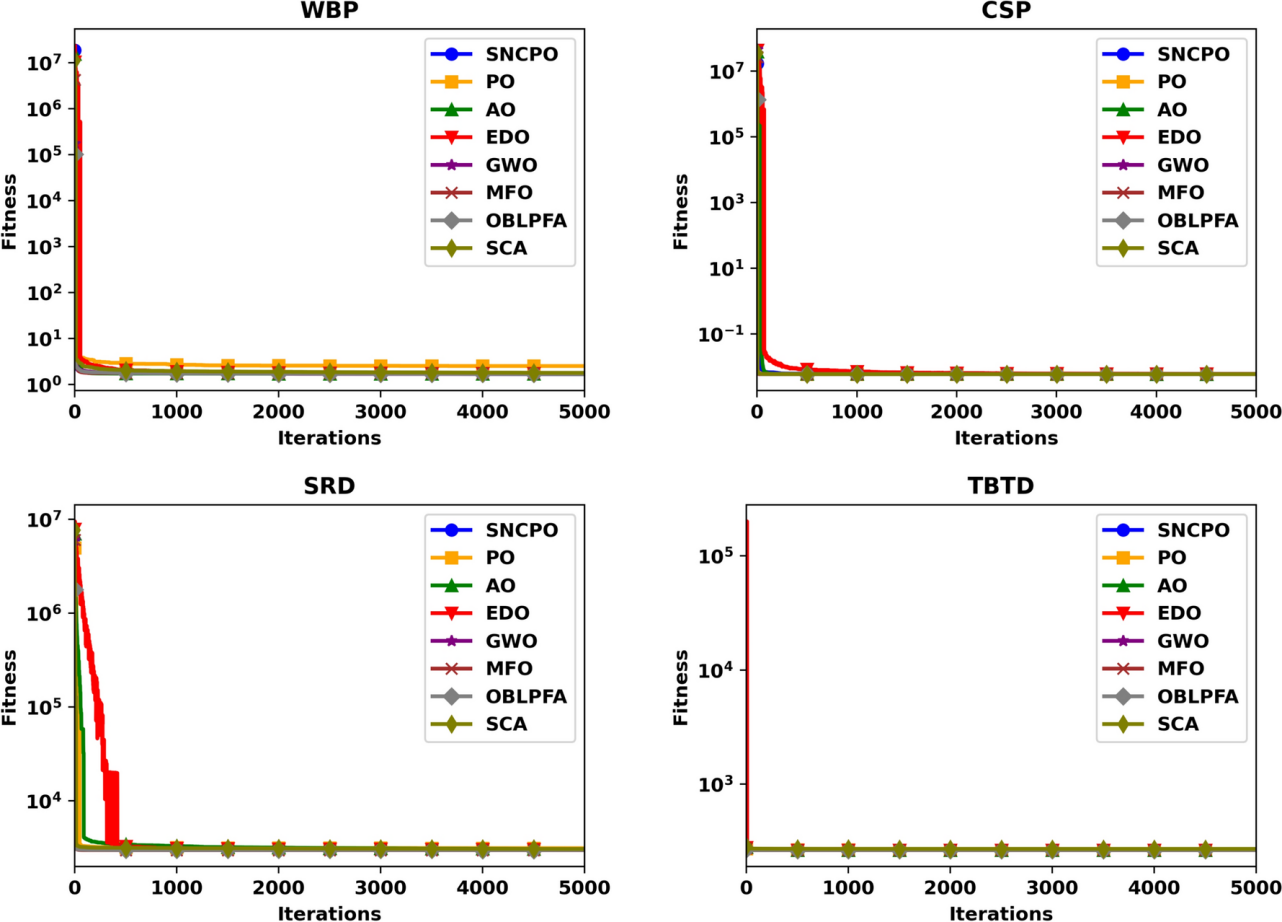
Convergence curve of SNCPO and other optimizers on engineering problems.

In the case of the TBTD problem, SNCPO exhibited highly competitive performance relative to other cutting-edge metaheuristic algorithms. Specifically, its results were on par with those achieved by AO, EDO, and OBLPFA, with SNCPO obtaining one of the best mean values for this problem. This further illustrates the algorithm’s robustness, demonstrating that SNCPO is well-equipped to handle engineering design problems characterized by complex nonlinear constraints and multiple interacting decision variables. The ability to maintain competitive performance in structural design optimization highlights the efficiency of SNCPO in balancing exploration and convergence precision. For the SRD problem, the experimental results indicate that MFO achieved the most optimal mean value. However, SNCPO still exhibited commendable competitiveness, achieving mean values that closely approached that of MFO. This finding aligns with the No Free Lunch (NFL) theorem, which postulates that no single optimization algorithm can consistently outperform all others across all problem types. The variation in performance across different problems reaffirms that different optimization strategies are more suited to specific problem structures. Despite MFO obtaining the best mean solution for SRD, SNCPO demonstrated a strong capacity to explore high-quality solutions, further establishing its credibility as a reliable engineering optimization algorithm. In addition to the numerical statistical analysis presented in Table 7, the convergence behavior of SNCPO, alongside the other compared algorithms, is depicted in Fig. 13. These convergence plots provide a visual representation of the solution progression over iterations, offering deeper insights into the search efficiency and optimization dynamics of each algorithm. The ability of SNCPO to converge rapidly toward high-quality solutions reinforces its practical applicability in engineering design problems, where computational efficiency and solution accuracy are of paramount importance.

### Experiments in ELM training

In this section, the parameter settings outlined in Table 1 are consistently applied across all competing optimization algorithms to ensure a fair and unbiased comparison. The number of iterations is set to 50, and the search range for determining the optimal weight and bias values of the ELM model is within the range of -10 to 10. To comprehensively evaluate the effectiveness of the SNCPO in optimizing the ELM model, 14 benchmark datasets obtained from the UCI Machine Learning Repository^66^ were utilized. Table 8 provides a detailed description of these datasets, capturing their key characteristics, such as the number of instances, attributes, and problem complexity. The experimental results, which summarize the accuracy performance in terms of the average (AVG) and standard deviation (STD) over 30 independent runs, are presented in Table 9. These results provide critical insights into the optimization effectiveness of SNCPO when employed in machine learning parameter tuning, particularly in training the ELM model. The findings indicate that the SNCPO consistently outperforms its counterparts, delivering superior accuracy across the majority of datasets. This underscores its robustness, stability, and efficiency in optimizing neural network parameters, which are crucial for ensuring high generalization performance in predictive modeling tasks. A more detailed analysis of Table 9 reveals that the SNCPO achieves superior average accuracy across most datasets compared to conventional optimization algorithms, including the PO, AO, EDO, MFO, OBLPFA, and SCA. The superior performance of SNCPO can be directly attributed to its innovative hybridization of competitive learning and the salp navigation strategy, which synergistically enhance its exploration and exploitation capabilities. By leveraging competitive learning, SNCPO facilitates improved solution refinement, ensuring that optimal weight and bias values are efficiently identified for ELM training. Simultaneously, the salp navigation strategy enables dynamic adjustments in the search process, preventing premature convergence and promoting global search efficiency.

**Table 8:** Description of data.

Datasets	Number of samples	Number of features	Number of labels
Appendicitis	106	7	2
Balance	625	4	3
Blood	748	4	2
BreastCancer	699	10	2
BreastEW	568	30	2
CongressEW	434	16	2
Digits	1797	64	10
Ecoli	336	7	7
Glass	214	10	6
Heart	270	13	2
HeartEW	270	13	2
Ionosphere	351	34	2
pathbased	300	2	3
Seeds	210	7	3

**Table 9:** Accuracy results using SNCPO and compared algorithm on ELM parameter optimization.

		**SNCPO**	**PO**	**AO**	**EDO**	**GWO**	**MFO**	**OBLPFA**	**SCA**
Appendicitis	AVG	**0.90045**	0.890417	0.883333	0.886458	0.876042	0.88125	0.888542	0.8875
	STD	**0.016984**	0.054683	0.041951	0.034125	0.030208	0.037567	0.027131	0.028175
Balance	AVG	0.931655	0.890544	0.930142	0.936879	**0.93818**	0.936288	0.933333	0.936525
	STD	0.012366	0.037102	0.007651	0.007611	**0.005815**	0.008939	0.009025	0.00787
Blood	AVG	**0.79522**	0.761333	0.770222	0.771259	0.772593	0.773778	0.772148	0.771259
	STD	0.009012	0.008152	0.007585	0.007961	0.007828	0.008842	0.006953	**0.006934**
BreastCancer	AVG	**0.975665**	0.904762	0.945079	0.948413	0.950159	0.946508	0.949206	0.948413
	STD	0.006262	0.057956	0.00919	0.007061	**0.004948**	0.008352	0.007776	0.004999
BreastEW	AVG	0.933585	0.907212	0.969201	0.972515	**0.982261**	0.971735	0.975244	0.97271
	STD	0.046364	0.054348	0.013113	0.013047	**0.008595**	0.012956	0.009238	0.011149
CongressEW	AVG	**0.952365**	0.889822	0.946819	0.943257	0.940712	0.936387	0.946819	0.943511
	STD	0.018322	0.040703	**0.009396**	0.012668	0.014338	0.012005	0.013501	0.013792
Digits	AVG	0.948512	0.91063	0.962136	0.958444	**0.974679**	0.958247	0.955679	0.950642
	STD	0.008548	0.017995	0.00379	0.00388	**0.003471**	0.0046	0.005772	0.004787
Ecoli	AVG	**0.968308**	0.934276	0.959925	0.958887	0.961056	0.958039	0.958982	0.959076
	STD	**0.003056**	0.015868	0.005523	0.004912	0.004232	0.006058	0.005413	0.004586
Glass	AVG	0.929679	0.895214	0.924274	0.933333	**0.948034**	0.934359	0.929744	0.927179
	STD	0.018576	0.025314	0.014833	0.01165	0.014721	0.018804	0.013229	**0.009869**
Heart	AVG	**0.874074**	0.733333	0.789712	0.77284	0.780658	0.783951	0.785597	0.778601
	STD	0.024868	0.059712	**0.019453**	0.025398	0.031151	0.03707	0.021608	0.028557
HeartEW	AVG	**0.864374**	0.739918	0.814815	0.797942	0.811934	0.809053	0.80535	0.80535
	STD	**0.017078**	0.06216	0.028222	0.026881	0.023189	0.024638	0.02522	0.028686
Ionosphere	AVG	0.850204	0.744969	0.817925	0.831132	**0.86195**	0.837421	0.828302	0.830503
	STD	0.026536	0.040487	0.033302	0.035327	**0.02254**	0.031397	0.034675	0.027477
Pathbased	AVG	0.978413	0.88321	0.970123	0.969383	**0.978519**	0.973827	0.975062	0.974568
	STD	0.026455	0.061074	0.015225	0.0186	0.016004	**0.013792**	0.017294	0.017917
Seeds	AVG	0.978382	0.957672	**0.992593**	0.989771	0.988007	0.988713	0.989771	0.989065
	STD	0.010909	0.049707	**0.006666**	0.008703	0.008806	0.008471	0.007353	0.009497

Significant values are given in bold.

Furthermore, the adaptability of SNCPO in handling machine learning optimization tasks is evident from its ability to maintain stable performance across multiple independent runs, as reflected in its relatively low STD values in Table 9. This stability is a key factor in real-world applications, where machine learning models require consistent optimization to deliver reliable predictions. The superior balance between exploration and exploitation achieved by SNCPO ensures that it effectively navigates complex search spaces, leading to optimal solutions that enhance the predictive accuracy of ELM. The experimental findings presented in this section affirm that the SNCPO is highly effective in machine learning parameter optimization. Its ability to outperform existing optimization techniques demonstrates its practical utility in training ELM models and, by extension, other machine learning architectures that rely on efficient parameter tuning. Given these promising results, future research could further explore the application of SNCPO in optimizing deep learning models, hyperparameter tuning, and feature selection, reinforcing its relevance in advancing the field of artificial intelligence.

## Conclusion and future work

In this study, we introduced the SNCPO, a novel hybrid metaheuristic algorithm designed to overcome the inherent limitations of the traditional PO. By integrating the pairwise competitive learning strategy from CSO with the adaptive navigation mechanism of the SSA, SNCPO effectively enhances population diversity, mitigates premature convergence, and achieves a well-balanced trade-off between exploration and exploitation. Extensive experimental evaluations on benchmark test functions (CEC2015 and CEC2020), real-world engineering optimization problems, and ELM training tasks demonstrated that SNCPO outperforms several contemporary optimization algorithms in terms of robustness, convergence speed, and solution accuracy. The results indicate that SNCPO consistently achieves superior optimization performance across various problem domains, reinforcing its potential as a powerful optimization tool.

Despite its notable advantages, SNCPO does present certain limitations. One of the primary challenges is its slightly higher computational cost compared to the traditional PO in a few optimization problems, which stems from its additional improvement mechanisms. Additionally, while SNCPO outperforms existing algorithms in most cases, a few benchmark functions indicate that further improvements in search efficiency are required to enhance its capability in highly complex landscapes. Future research directions will focus on enhancing the scalability of SNCPO for high-dimensional optimization problems and exploring its applicability in more advanced machine learning models beyond ELM. Further developments may include parallelized implementations to improve computational efficiency and adaptability. Moreover, extending the SNCPO framework to domains such as feature selection, image processing, and renewable energy optimization could further underscore its versatility and real-world applicability. These advancements will contribute to solidifying SNCPO’s role as a state-of-the-art optimization approach for solving complex, multidimensional problems across various scientific and engineering disciplines.

## Data Availability

The data obtained through the experiments are available upon request from corresponding author. To protect study participant privacy, the data will be available upon request.
